# Comparative Analysis of Immune Checkpoint Molecules and Their Potential Role in the Transmissible Tasmanian Devil Facial Tumor Disease

**DOI:** 10.3389/fimmu.2017.00513

**Published:** 2017-05-03

**Authors:** Andrew S. Flies, Nicholas B. Blackburn, Alan Bruce Lyons, John D. Hayball, Gregory M. Woods

**Affiliations:** ^1^Menzies Institute for Medical Research, University of Tasmania, Hobart, TAS, Australia; ^2^Department of Pharmacy and Medical Sciences, University of South Australia, Adelaide, SA, Australia; ^3^School of Medicine, South Texas Diabetes and Obesity Institute, University of Texas Rio Grande Valley, Brownsville, TX, USA; ^4^School of Medicine, University of Tasmania, Hobart, TAS, Australia; ^5^Discipline of Obstetrics and Gynaecology, School of Medicine, Robinson Research Institute, The University of Adelaide, Adelaide, SA, Australia

**Keywords:** devil, transmissible tumor, cosignaling immunotherapy, checkpoint blockade, wild immunity, allograft, transplant rejection, evolution

## Abstract

Immune checkpoint molecules function as a system of checks and balances that enhance or inhibit immune responses to infectious agents, foreign tissues, and cancerous cells. Immunotherapies that target immune checkpoint molecules, particularly the inhibitory molecules programmed cell death 1 and cytotoxic T-lymphocyte-associated protein 4 (CTLA-4), have revolutionized human oncology in recent years, yet little is known about these key immune signaling molecules in species other than primates and rodents. The Tasmanian devil facial tumor disease is caused by transmissible cancers that have resulted in a massive decline in the wild Tasmanian devil population. We have recently demonstrated that the inhibitory checkpoint molecule PD-L1 is upregulated on Tasmanian devil (*Sarcophilus harrisii*) facial tumor cells in response to the interferon-gamma cytokine. As this could play a role in immune evasion by tumor cells, we performed a thorough comparative analysis of checkpoint molecule protein sequences among Tasmanian devils and eight other species. We report that many of the key signaling motifs and ligand-binding sites in the checkpoint molecules are highly conserved across the estimated 162 million years of evolution since the last common ancestor of placental and non-placental mammals. Specifically, we discovered that the CTLA-4 (MYPPPY) ligand-binding motif and the CTLA-4 (GVYVKM) inhibitory domain are completely conserved across all nine species used in our comparative analysis, suggesting that the function of CTLA-4 is likely conserved in these species. We also found that cysteine residues for intra- and intermolecular disulfide bonds were also highly conserved. For instance, all 20 cysteine residues involved in disulfide bonds in the human 4-1BB molecule were also present in devil 4-1BB. Although many key sequences were conserved, we have also identified immunoreceptor tyrosine-based inhibitory motifs (ITIMs) and immunoreceptor tyrosine-based switch motifs (ITSMs) in genes and protein domains that have not been previously reported in any species. This checkpoint molecule analysis and review of salient features for each of the molecules presented here can serve as road map for the development of a Tasmanian devil facial tumor disease immunotherapy. Finally, the strategies can be used as a guide for veterinarians, ecologists, and other researchers willing to venture into the nascent field of wild immunology.

## Introduction

The immune system plays a vital role in protecting an animal from pathogens and cancers through various immunosurveillance and effector mechanisms. The pathogenic cells and/or microbes that escape or neutralize host immunity produce more surviving descendants that are in turn more effective at evading immune responses. The architecture of the vertebrate immune system and the mechanisms by which immune responses are regulated are the result of this long and complex evolutionary arms race. This arms race drives continuous evolution of the immune system, but many defenses have persisted throughout this long-history and are conserved across speciation events.

**Figure 1:**
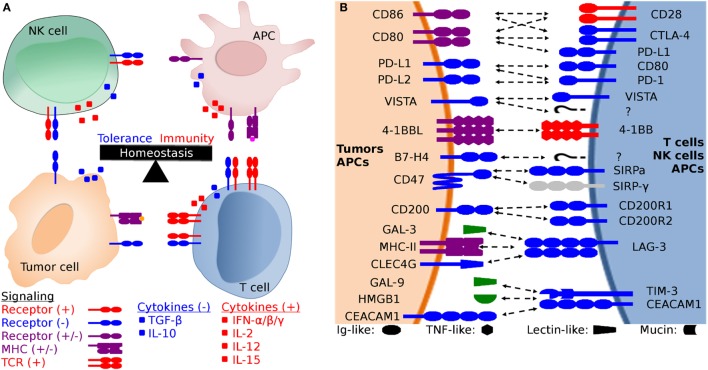
**Generalized schematic of checkpoint molecule structure and expression, and the balance between stimulatory and inhibitory signaling**. **(A)** The net balance of inhibitory (−, blue) and stimulatory (+, red) signals regulates antitumor immune responses. The tumor microenvironment is dynamic and is molded by inhibitory and stimulatory signals both in the microenvironment and distant tissues (i.e., lymph nodes). Development of cytotoxic effector T cells and NK cells is regulated by the balance between inhibitory and stimulatory signaling, including both receptor:coreceptor interactions and cytokines. Both receptor and cytokine expression are also regulated by that balance of stimulatory and inhibitory signals, creating feedback loops that evolved to eliminate pathogens and cancer while simultaneously limiting collateral damage. **(B)** Expanded view of potential receptor:coreceptor interactions between T cells, NK cells, antigen-presenting cells (APCs), and tumor cells. The graphic here demonstrates some of the best characterized pathways, with a particular focus on T cell and NK cell interactions with tumor cells and APCs. Note that many of the ligands depicted here are expressed on several cell types and interactions with coreceptors and ligands can result in different functional outcomes depending on the cell types and microenvironment. For example, CD47 is expressed on all cells, but the pathway of primary interest for transmissible tumors is for the ability of tumor cell-expressed CD47 to inhibit phagocytosis *via* binding to SIRP-α on APCs. In addition to binding to SIRP-α, CD47 also binds to SIRP-β, SIRP-γ, THBS1, and THBS2, but we have only shown the CD47:SIRP-α interaction here to keep the figure relatively simple. Both V-domain Ig suppressor of T cell activation (VISTA) and B7-H4 have unknown coreceptors (labeled as “?” in the diagram) whose function has been studied using fusion proteins, but the genes for the coreceptors have yet to be identified.

Immune checkpoint molecules function as a complex system of checks and balances that promote or inhibit immune responses. Checkpoint molecules likely evolved to precisely balance the need to control pathogens and cancer with the need to maintain tolerance to healthy tissues and the microbiota (Figure 1). However, the delicate balance of tolerance and immunity can be upset by pathogens and tumor cells that subvert inhibitory pathways to evade host immune defenses.

Checkpoint molecule immunotherapy has recently surged onto the world stage, but this field emerged over three decades of basic immunobiological research, primarily focused on T cells. In general, activation of T cells requires signaling through the T cell receptor (TCR) (signal 1) and cosignaling through CD28 (signal 2), a constitutively expressed disulfide-linked homodimer on the surface of naïve T cells (1–3). Signal one is derived from interactions of the TCR with its cognate major histocompatibility complex (MHC) peptide-complex (4). Signal two is derived from CD28 binding with either CD80 (B7-1) or CD86 (B7-2), primarily expressed by dendritic cells (DCs) and other antigen-presenting cells (APCs), such as macrophages and B cells. TCR signaling in the absence of CD28 cosignaling can result in T cell anergy (5). Following activation of T cells, the inhibitory molecule cytotoxic T-lymphocyte-associated protein 4 (CTLA-4) is upregulated on the cell surface (6). CTLA-4 has a higher affinity for both CD80 and CD86 than does CD28 (7–9), and thus CD28 cosignaling is suppressed by CTLA-4 outcompeting CD28 for CD80 and CD86 binding. The result is a downregulation of the T cell response and loss of T cell function.

Immunotherapies targeting the CTLA-4 checkpoint molecule have opened new doors in the realm of human oncology. Two antagonistic anti-CTLA-4 mAbs, ipilimumab (IgG1 isotype) and tremelimumab (IgG2 isotype), that block interactions between CTLA-4 and its coreceptors CD80 and CD86 have been clinically approved for advanced stage cancers. The exact mechanisms by which anti-CTLA-4 works are not completely understood. Accumulating evidence suggests that anti-CTLA-4 works primarily in the secondary lymphoid organs by releasing pre-existing T cells from inhibitory signaling to target tumor neoantigens and by depleting Tregs in the tumor microenvironment (10, 11).

Despite the promising early results of anti-CTLA-4 mAb monotherapy in human clinical trials, objective response rates remained low in most cases. Improvements have been observed when anti-CTLA-4 was paired with other treatments, such as the chemotherapy drug dacarbazine (12). However, the most promising outcomes have been observed when anti-CTLA-4 mAb is paired with blockade of the programmed cell death 1 (PDCD1, PD-1) protein (13–15).

Anti-PD-1 has been the most successful checkpoint blocking monoclonal antibody in treating human cancer, in monotherapies or in combination therapies. Anti-CTLA-4 and anti-PD-1 combination therapies have achieved unprecedented objective response rates of nearly 60% in patients with stage III and IV melanoma (14, 15). PD-1 immunotherapies have focused largely on stimulating T cell antitumor responses by blocking binding of PD-1 to its ligands PD-L1 (B7-H1, CD274) and PD-L2 (B7-DC, CD273). PD-1 blockade abrogates the inhibitory effects of PD-1 ligation mediated by the immunoreceptor tyrosine-based inhibitory motif (ITIM) and immunoreceptor tyrosine-based switch motif (ITSM) in the PD-1 cytoplasmic tail (16–18). In addition to blocking the inhibitory effects of PD-1, blockade of PD-L1 ligation can render tumor cells less resistant to apoptosis (19). PD-L1 and PD-L2 proteins are largely absent in normal tissues, but PD-L1 expression is elevated in more than 20 types of human cancer [reviewed in Ref. (20)]. Furthermore, many cancers and healthy cells upregulate PD-L1 expression in response to inflammatory cytokines, such as interferon-gamma (IFN-γ).

In humans, PD-L1 expression in the tumor microenvironment is associated with an inhibited T cell antitumor response and reduced patient survival [reviewed in Ref. (20)]. PD-L1 expression in the tumor microenvironment plays an important role in shaping a dysfunctional immune response through reprogramming of APCs, regulatory T cells, and expression of inhibitory molecules. PD-L2 is not routinely detected on cancer cells, but is commonly expressed by DCs, which led to the alternative name of B7-DC (21). A second coreceptor for PD-L2, repulsive guidance molecule b, has recently been discovered and has been implicated in respiratory immune tolerance (22).

Orthologs of CTLA-4 and PD-1 and their coreceptors have been found in nearly all mammals and birds studied to date (23, 24), but comparatively little is known about the potential for checkpoint molecule immunotherapy in the field of veterinary medicine or in the realm of wildlife disease. Initial investigations into the function of CTLA-4, PD-1, and PD-L1 have been undertaken for canids (25–30), felids (31), and bovids (32–35), and results suggest that the CTLA-4 and PD-1/PD-L1 expression patterns and function are similar among these placental mammals.

An area where checkpoint molecule immunology could make a wildlife conservation impact is in the unusual cases of transmissible tumors. These transmissible tumors represent a unique immunological enigma because they can also simultaneously be considered infections and allografts. Disease progression, transmission, and the host immune response can only be properly understood when these tumors are considered in this complex tumor–infection–allograft context.

The Tasmanian devil facial tumor (DFT) was identified in 1996, and in 2006 the etiologic agent was found to be cancer cells that are transmitted between biting devils (36, 37). At the time of discovery, the only other known naturally transmissible tumors were the canine (*Canis lupus familiaris*) transmissible venereal tumor (38, 39) and a set of related Syrian hamster (*Mesocricetus auratus*) transmissible sarcomas (40, 41). In recent years, additional transmissible tumors have been discovered in soft-shell clams and other bivalves (42, 43). Remarkably, in 2015, we discovered a second transmissible devil facial tumor (DFT2) in wild Tasmanian devils (referred to as devils hereafter) that is genetically distinct from the original devil facial tumor (DFT1) (44).

Previous research has shown that reduced cell surface expression of MHC-I plays a major role in DFT1 immune evasion, but that IFN-γ can upregulate beta-2 microglobulin (β2m), ergo MHC-I expression (45). This immune evasion mechanism has been described in many types of cancer, and begs the question as to why the MHC-negative DFT cells are not targeted by NK cells in line with the “missing-self” hypothesis (46). Studies have demonstrated the NK cell genes are present in devils and that devil NK-like cells can mount an antitumor response (47–49). Despite the complimentary mechanisms of cytotoxic T cells and NK cells, immune responses against the DFTs are rare (50), suggesting that additional immune evasion mechanisms could be present.

Recent vaccination efforts have demonstrated that IFN-γ-stimulated DFT cells can induce antitumor responses and tumor regressions in a subset of cases (51). However, initial forays into checkpoint molecule immunology of the DFT disease has shown that both DFT1 and DFT2 also upregulate PD-L1 upon exposure to IFN-γ (52), suggesting that anti-DFT immunotherapies could be enhanced by neutralizing the PD-1 inhibitory pathway. Given that the immune system has many redundant mechanisms for identifying and killing infectious agents and tumors, it is likely that the transmissible tumors employ additional methods beyond MHC-I downregulation and PD-L1 upregulation for evading host immune defenses and facilitating tumor transmission.

We have performed a comparative analysis of the protein sequences of key immune checkpoint molecules in the three mammalian species known to harbor naturally transmissible cancers (devils, dogs, and hamsters) and six other mammals (Table 1). We have not included the analysis of PD-1, PD-L1, and PD-L2 here, as we have previously published this information (52). Understanding the broad role of checkpoint molecules in these complex diseases has the potential to shed light on fundamental aspects of immune evasion, transplant tolerance and rejection, and infectious disease; this is logically relevant to both human and veterinary medicine. This checkpoint molecule analysis and review of salient features for each of the molecules presented here can serve as road map for the development of a DFT immunotherapy and as a guide for veterinarians, ecologists, and other researchers willing to venture into the nascent field of wild immunology.

**Table 1:** **Names and identifiers for species used in comparative sequence analysis**.

#	Short name	Common name	Species name	Taxid
1	Devil	Tasmanian devil	*Sarcophilus harrisii*	9305
2	Opossum	Gray short-tailed opossum	*Monodelphis domestica*	13616
3	Bat	Little brown bat	*Myotis lucifugus*	59463
4	Cat	Domestic cat	*Felis catus*	9685
5	Cattle	Cattle	*Bos taurus*	9913
6	Dog	Domestic dog	*Canis familiaris*	9615
7	Hamster	Golden hamster	*Mesocricetus auratus*	10036
8	Human	Human	*Homo sapiens*	9606
9	Mouse	House mouse	*Mus musculus*	10090

## Materials and Methods

### Species

We included nine species in our comparative analysis of checkpoint molecules. Humans and mice were included because checkpoint molecules have been best characterized in these species. We have included the little brown bat (*Myotis lucifugus*) in our comparative analyses because like devils, the species has recently undergone a dramatic population crash due to disease, and understanding bat immunology has become increasingly important due to the ability of bats to serve as reservoirs for viral pathogens (e.g., rabies, ebola, SARS). We have also included cats (*Felis catus*) and cattle (*Bos taurus*) due to their widespread distribution as important domestic animals. See Table 1 for a full list of species names and abbreviations used.

### Sequences

DNA sequences for all genes analyzed here were collected from GenBank and Ensembl and reference numbers for all sequences are available in Table S1 in Supplementary Material (53). All reference sequences current as of 30-Jan-2017, including the most current devil genome (Devil_ref v7.0). The complete coding sequence for the V-domain Ig suppressor of T cell activation (VISTA), CD47, and TIM-3 genes was not available from either GenBank or Ensembl, so sequences were obtained from a *de novo* transcriptome assembly (Table S2 in Supplementary Material) described below. The full coding sequences were submitted to GenBank with accession numbers: KY856749 (VISTA), KY856750 (CD47), and KY856751 (TIM-3). Where more than one sequence was present we generally chose GenBank transcript X1, unless preliminary alignments suggested that an alternative transcript was a better match to the other species used here. BLAST was used to find additional potential matching sequences for each gene (54). The available reference sequences for several genes that have not been characterized in their respective species contained elongated 5′ regions upstream of the likely start codon, so we manually truncated the amino acid sequences depicted in the alignment figures for the following genes: opossum CD80 and VISTA, bat CD86 and CD47, and hamster B7-H4.

### RNA Sequencing (RNA-seq)

RNA sequencing data were generated as part of a previous study (55). Briefly, RNA was extracted from peripheral blood mononuclear cells (PBMCs) obtained from two disease-free devils. Illumina single-end 100 bp RNA-seq was conducted by the Australian Genome Research Facility. Sequencing generated 82,609,965 and 82,254,904 sequences, respectively. Fastq reads for each sample were assessed with FastQC version 0.11.5 (56), with both samples having acceptable per base sequence quality measurements. Genome-independent *de novo* assembly of the RNA-seq data was completed using the Trinity platform version 2.2.0 (57, 58) with default settings applied. This generated a reference transcriptome of assembled transcripts collectively from the two samples. The *de novo* transcriptome assembly was queried with BLASTN version 2.4.0+ (59) with coding sequences of the target genes obtained from Ensembl (53), database version 87.7, and UCSC Genome Browser (60). This identified transcript sequences from the assembled transcriptome of our target genes to use in comparative alignment analysis. Further information on this analysis is detailed in the Data Sheet S1 in Supplementary Material. Access to the raw fastq sequence data, Trinity assembled *de novo* transcriptome, and BLASTN results are detailed in the Data Sheet S1 in Supplementary Material.

### Protein Sequence Alignments

Protein sequences were aligned with CLC sequence viewer using a progressive alignment algorithm with gap open cost = 5, gap extension cost = 1, end gap cost = free (61, 62). Pairwise protein sequence identities between devils and other species were determined using the default parameters on the Clustal Omega web server (63). We used the RasMol coloring scheme to highlight traditional amino acid properties in all sequence alignments, which aids in interpretation of amino differences across species (64).

### Analysis of Protein Sequences

All sequences were analyzed using the ExPASy server ProtParam tool to predict molecular weight and extinction coefficients (65). The eukaryotic linear motif (ELM) tool was used to predict key signaling motifs (66). The Simple Modular Architecture Research Tool (SMART) and the Phobius web server (67) were used to predict Ig-like domains and membrane topology (68). SignalP was used to confirm predicted signal peptides (69). Disulfide bonds and other unique features of each protein that were annotated on UniProt (70) were used to predict features in devil protein sequences.

## Results

The CTLA-4 and CD28 pathways are perhaps the most studied of the checkpoint molecules, but functional testing in species other than mice and humans is limited. A battery of tools (i.e., recombinant proteins, monoclonal antibodies) and time are needed for proper testing of checkpoint molecule function, but much information and insight into function can be gained by analysis of protein sequences. For each molecule analyzed, we begin with a description of the general structure (i.e., Ig-like superfamily domains) and proceed to more nuanced details. This provides insight into potential functions of the molecules and can provide direction for designing critical experiments prior to the embarking on the time-consuming process of empirical testing of molecular function. See Table S3 in Supplementary Material for details of checkpoint molecule structure.

### CTLA-4, CD28, CD80, and CD86

**Figure 2:**
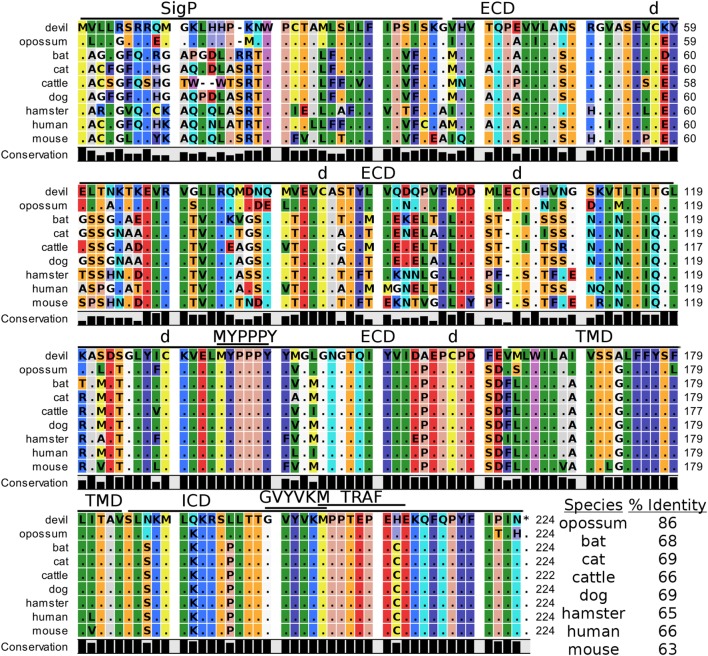
**Alignment of cytotoxic T-lymphocyte-associated protein 4 reference genes for nine species**. The predicted signal peptides (SigP), extracellular domain (ECD), transmembrane domain (TMD), and intracellular domain (ICD) are demarcated with bars. “d” above the alignment represents predicted disulfide bonds. MYPPPY indicates the conserved CD80- and CD86-binding site. GVYVKM indicates a conserved cytoplasmic inhibitory domain and TRAF indicates a putative TRAF6-binding site. The black bars graphs below the alignment represent the conservation of amino acids across all nine species. The percent amino acid sequence identity between devils and other species is shown in the bottom right corner of the alignment.

**Figure 3:**
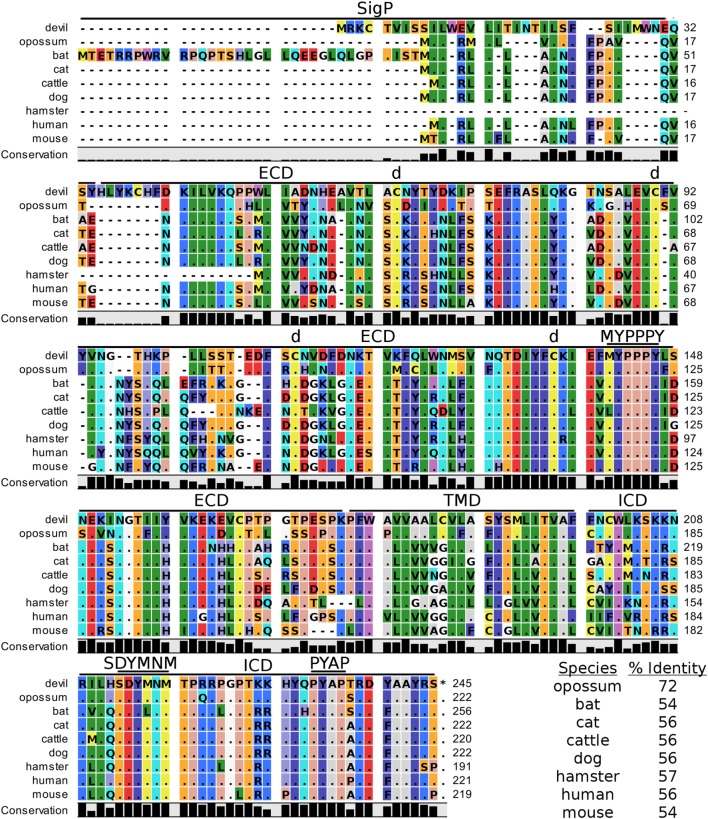
**Alignment of CD28 reference genes for nine species**. The predicted signal peptides (SigP), extracellular domain (ECD), transmembrane domain (TMD), and intracellular domain (ICD) are demarcated with bars. “d” above the alignment represents predicted disulfide bonds. MYPPPY indicates the conserved CD80- and CD86-binding site, and SDYMNM and PYAP indicate key phosphorylation sites involved with cell proliferation and activation. The black bar graphs below the alignment represent the conservation of amino acids across all nine species. The percent amino acid sequence identity between devils and other species is shown in the bottom right corner of the alignment.

**Figure 4:**
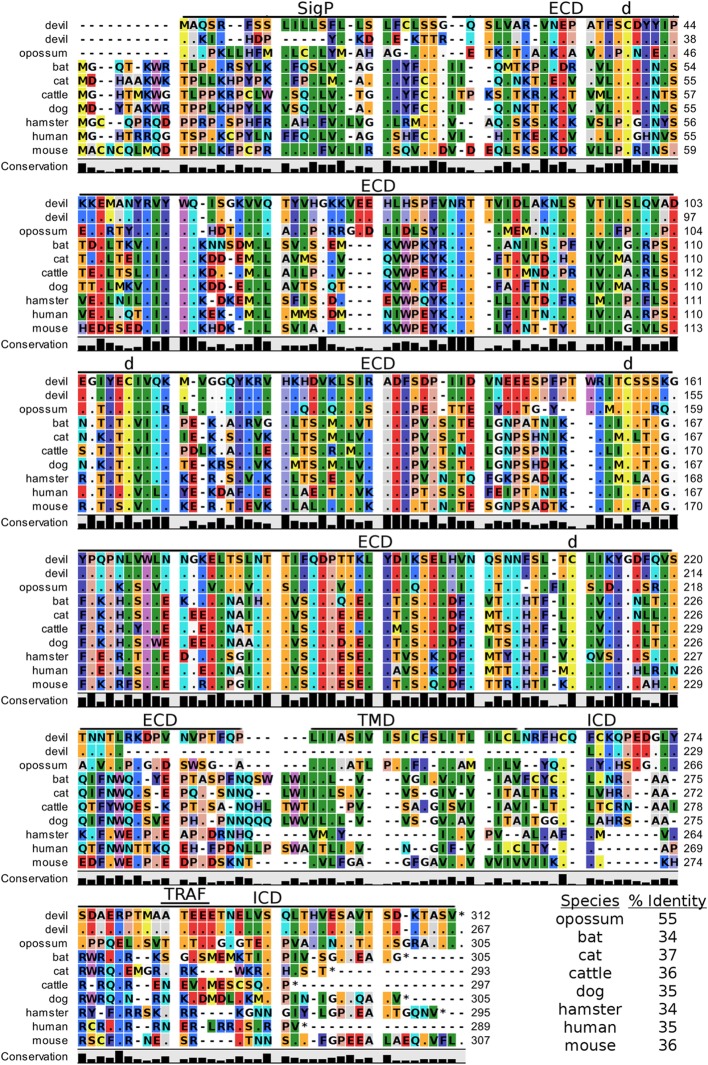
**Alignment of CD80 reference genes for nine species**. The predicted signal peptides (SigP), extracellular domain (ECD), transmembrane domain (TMD), and intracellular domain (ICD) are demarcated with bars. The uppermost devil CD80 sequence is the full-length sequence and the lower devil CD80 is the potential secreted CD80 sequence. “d” above the alignment represents predicted disulfide bonds. TRAF indicates a putative TRAF1/2-binding site. The black bar graphs below the alignment represent the conservation of amino acids across all nine species. The percent amino acid sequence identity between devils and other species is shown in the bottom right corner of the alignment.

**Figure 5:**
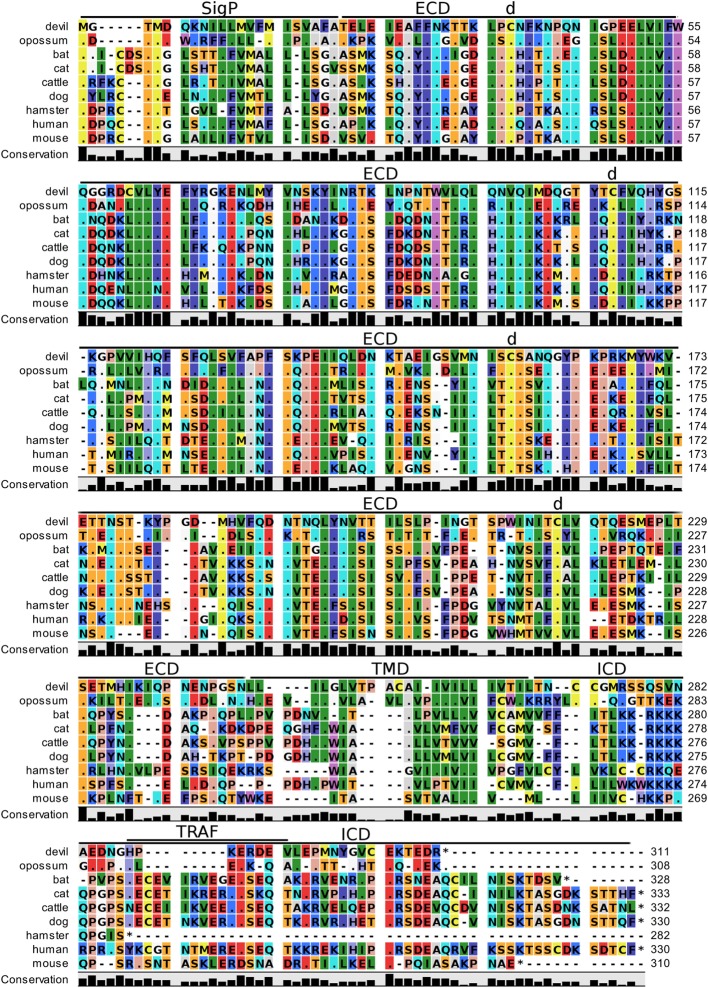
**Alignment of CD86 reference genes for nine species**. The predicted signal peptides (SigP), extracellular domain (ECD), transmembrane domain (TMD), and intracellular domain (ICD) are demarcated with bars. “d” above the alignment represents predicted disulfide bonds. TRAF indicates a putative TRAF6-binding site. The black bar graphs below the alignment represent the conservation of amino acids across all nine species. The percent amino acid sequence identity between devils and other species is shown in the bottom right corner of the alignment. See Table 2 for the sequence identity between devils and other species.

Devil CTLA-4 and CD28 are predicted to be 25 and 28 kDa, respectively, type-I transmembrane proteins with a single Ig-like V-type extracellular domain (ECD) (Figure 1). Protein sequence identity for CTLA-4 between devils and opossums was 86% and ranged from 63 to 69% between devils and placental mammals (Figure 2; Table 2). Percent identity for CD28 among species was lower than for CTLA-4, with 72% between devils and opossums and ranging from 54 to 57% between devils and placental mammals (Figure 3). Devil CTLA-4 had approximately 29% amino acid sequence identity with CD28. The CTLA-4 and CD28 coreceptors CD80 and CD86 are predicted to both be 35 kDa type-I transmembrane proteins that contain Ig-like V-type and an Ig-like C2-type domains. Devil CD80 and CD86 share only 21% sequence identity with each other, and the identity across the species analyzed here ranges from 34 to 55% and 32–49% for CD80 (Figure 4; Table 2) and CD86 (Figure 5; Table 2), respectively; the percent identity for CD80 and CD86 across species is the lowest of any of the checkpoint molecules compared here. All intra- and intermolecular disulfide bonds that have been identified in humans for CTLA-4, CD28, CD80, and CD86 are likely present in all the orthologs analyzed here, as we observed 100% conservation of cysteine residues at these sites in all species tested (Figures 2–5).

**Table 2:** **Protein sequence percent identity between checkpoint molecules in devils and eight other species**.

Species	Cytotoxic T-lymphocyte-associated protein 4	CD28	CD80	CD86	V-domain Ig suppressor of T cell activation	4-1BB	CD47	SIRP-α	CD200	TIM-3	Lymphocyte activation gene 3	B7-H4
Opossum	85.7	72.4	55.2	49.2	73.7	75.5	87.1	69.2	65.7	52.7	72.5	93.9
Bat	68.5	53.9	33.9	35.9	65.6	48.0	54.2	56.3	54.7	43.0	48.7	84.1
Cat	68.9	55.9	36.9	35.1	64.8	51.2	65.2	56.6	56.3	43.3	46.3	85.9
Cattle	66.1	56.0	36.3	35.9	65.8	50.8	67.4	55.3	53.2	42.1	50.6	85.9
Dog	68.9	56.4	35.0	37.8	63.2	53.8	67.1	56.7	52.8	43.1	43.0	87.7
Hamster	65.3	57.4	33.6	32.4	60.0	43.4	64.2	52.7	51.7	44.4	48.7	79.6
Human	65.8	56.2	35.5	36.6	64.9	52.2	66.8	55.8	56.2	43.2	51.9	87.0
Mouse	63.1	54.4	35.7	35.0	61.9	44.2	64.5	53.1	53.2	42.3	50.0	81.9

*Percent identity was calculated by Clustal 2.1 algorithm*.

#### Key Ligand-Binding Regions Conserved in Devil CTLA-4 and CD28

Our analysis shows that devil CTLA-4 and CD28 both contain the MYPPPY motif that is necessary for binding of CTLA-4 and CD28 to CD80 and CD86 in humans and mice (Figures 2 and 3) (7). Of the 18 combined CTLA-4 and CD28 sequences aligned here, only cattle CD28 did not contain the CD28_MYPPPY_ motif (71); cattle had a CD28_LYPPPY_ sequence, which matches the CD28_LYPPPY_ and CTLA-4_LYPPPY_ that has been previously reported in pigs (*Sus scrofa*) and decreases affinity of porcine CTLA-4 for human CD80 and CD86 (35, 72). Additionally, all species also contain the CTLA_GVYVKM_ inhibitory domain. Except for bats, all species also contain the key CD28_SDYMNM_ and CD28_PYAP_ motifs are critical for intracellular signaling that promotes T cell proliferation and survival *via* the CD28–PI3K–AKT [reviewed in Ref. (73)]. Bats have a CD28_M228L_ substitution in the CD28_SDYMNM_ motif.

#### Potential Unique Signaling Motifs Identified

The two marsupials examined contain a putative TRAF6-binding domain in CTLA-4 proteins that was not present in any of the placental mammals. We identified a putative ITIM in predicted the ECD of dog CD80_VKYGDL_ and a TRAF1/2-binding site in the devil CD80_ATEEE_ cytoplasmic domain (Figure 4). Our analysis predicted a TRAF6-binding site in the cytoplasmic tail of devil CD86_GHPKERDEV_, a motif not predicted for CD86 in other species (Figure 5). Interestingly, the ELM tool predicted a putative ITIM domain in the second Ig-like segment of the human CD86_IEYDGI_ ECD. To our knowledge, this putative ITIM has not been previously reported, and thus the potential inhibitory capacity of this motif is unknown.

### V-Domain Ig Suppressor of T Cell Activation

**Figure 6:**
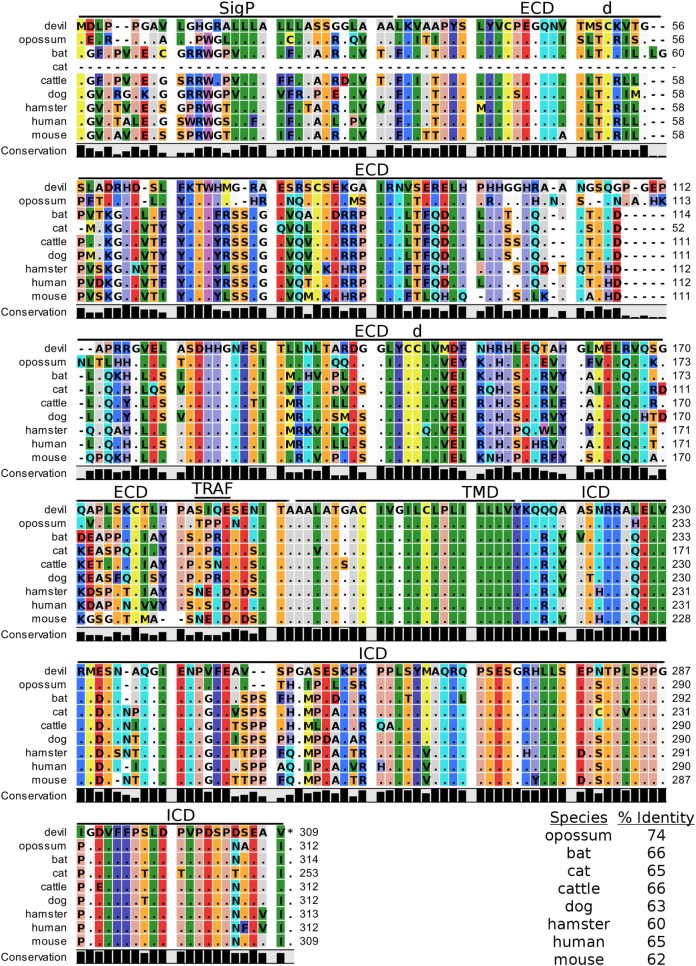
**Alignment of V-domain Ig suppressor of T cell activation reference genes for nine species**. The predicted signal peptides (SigP), extracellular domain (ECD), transmembrane domain (TMD), and intracellular domain (ICD) are demarcated with bars. “d” above the alignment represents predicted disulfide bonds and “TRAF” indicates a potential TRAF1/2-binding site. The black bar graphs below the alignment represent the conservation of amino acids across all nine species. The percent amino acid sequence identity between devils and other species is shown in the bottom right corner of the alignment.

Devil VISTA (PD-1H, C10orf54, Gi24, DD1α, Dies1) is a type-I transmembrane protein in the Ig superfamily predicted to be 33 kDa. The full coding sequence for this gene was previously not available in the devil genome (Devil_ref v7.0), but our *de novo* RNA transcriptome assembly uncovered a full coding devil VISTA transcript. Devil VISTA shares 60–74% sequence identity at the protein level with the other species analyzed here (Figure 6; Table 2). The ELM tool predicted a TRAF1/2-binding site in the ECD of devil VISTA_SIQE_.

### 4-1BB

**Figure 7:**
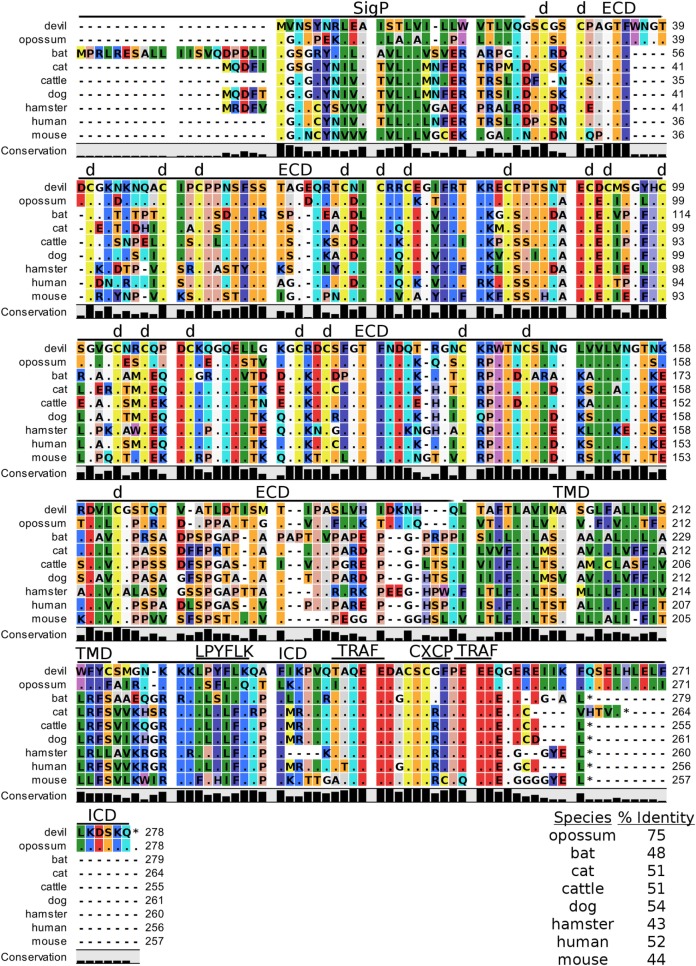
**Alignment of 4-1BB reference genes for nine species**. The predicted signal peptides (SigP), extracellular domain (ECD), transmembrane domain (TMD), and intracellular domain (ICD) are demarcated with bars. “d” above the alignment represents predicted disulfide bonds. Two potential TRAF1/2-binding sites are demarcated by “TRAF.” “CXCP” marks a motif associated with optimal T cell response in mice, and “LPYFLK” denotes a potential inhibitory domain in devils. The black bar graphs below the alignment represent the conservation of amino acids across all nine species. The percent amino acid sequence identity between devils and other species is shown in the bottom right corner of the alignment.

4-1BB (TNFRSF9, CD137) is a predicted 31 kDa member of the tumor necrosis factor (TNF) receptor family expressed predominantly as a type-I transmembrane homodimer (74, 75). 4-1BB ligand (4-1BBL, TNFSF9, CD137L) is a TNF family member of 20 kDa, expressed as a type II transmembrane homotrimer (Figure 1). SMART analysis of devil 4-1BB identified two TNFR-Cys repeat domains, whereas human 4-1BB contains four TNFR-Cys repeats (Figure 7). All cysteine repeats in the four human TNFR-Cys domains were conserved in devils, suggesting that devil 4-1BB might also contain four TNFR-Cys domains despite not being predicted by the SMART program. Indeed, all 20 cysteine residues involved in disulfide bonds in human 4-1BB were also present in devil 4-1BB. Overall sequence identity among species compared here ranged from 43 to 75%, with mouse and hamster 4-1BB having the lowest percent identities with devil 4-1BB (Table 2).

We identified a putative inhibitory domain in devil 4-1BB_LPYFLK_ based on alignment with a potential inhibitory domain in human 4-1BB_LLYIFK_ (76, 77). The putative inhibitory domain resembles an ITIM domain but lacks the I, L, or V in the terminal amino acid position. To date, this potential inhibitory domain has only been reported in humans, but several species analyzed here have similar motifs. Additionally, mice have a 4-1BB_CXCP_ motif in the cytoplasmic domain that facilitates binding of CD4 and CD8 molecules to p56^lck^, a protein tyrosine kinase that is important for optimal T cell responses (78, 79); of the nine species compared here, mice are the only species that have the 4-1BB_CXCP_ sequence. Our analysis using the ELM tool predicted two TRAF1/2 ligand-binding sites (devil 4-1BB_TAQEE_, 4-1BB_PEEE_), which align with the known TRAF1/2-binding sites in human and mouse 4-1BB (80).

### CD47

**Figure 8:**
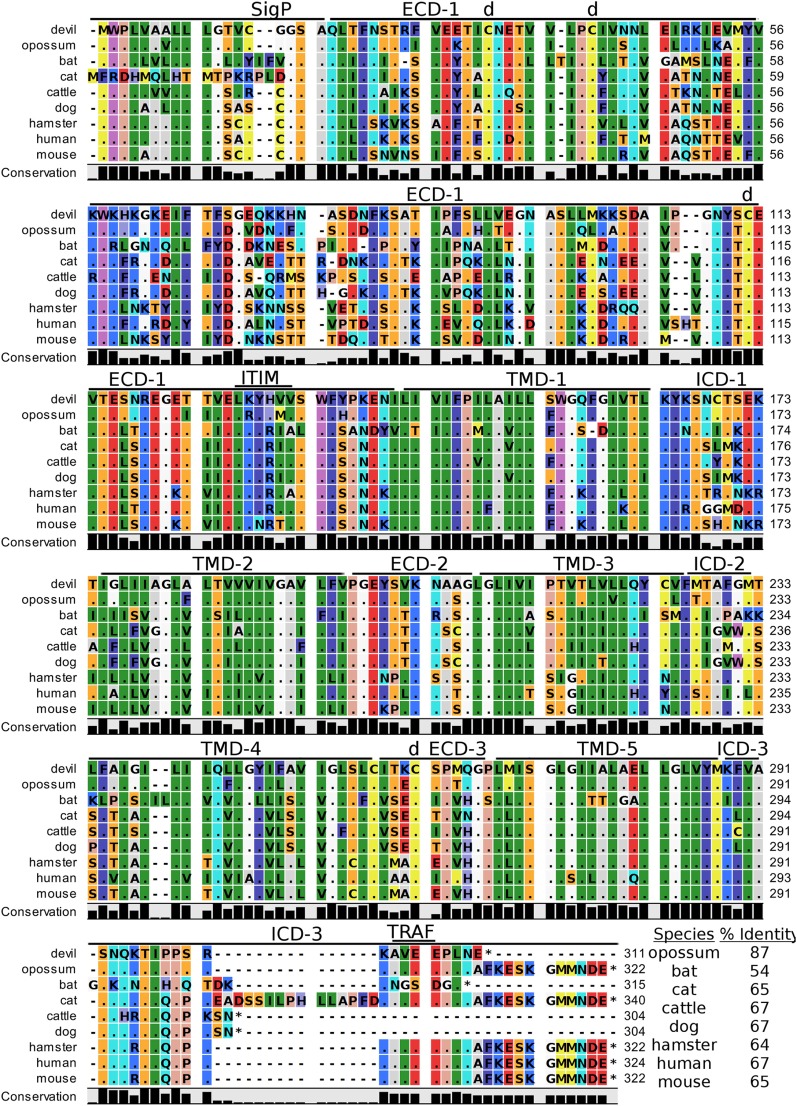
**Alignment of CD47 reference genes for nine species**. The predicted signal peptides (SigP), extracellular domains (ECDs), transmembrane domains (TMDs), and intracellular domain (ICD) are demarcated with bars. “d” above the alignment represents predicted disulfide bonds. “TRAF” indicates a potential TRAF1/2-binding site and immunoreceptor tyrosine-based inhibitory motif (ITIM) marks a putative ITIM motif predicted in the ECD. The black bar graphs below the alignment represent the conservation of amino acids across all nine species. The percent amino acid sequence identity between devils and other species is shown in the bottom right corner of the alignment.

Human CD47, also known as integrin-associated protein for its role in cell adhesion, is a cell surface protein that contains five transmembrane segments and a single Ig-like V-type domain (81, 82). The most up-to-date gene sequences for devil CD47 in Ensembl (ENSSHAT00000014546.1) did not contain a full coding sequence (i.e., no start codon) and the GenBank (XM_012544496) gene had an extended 5′ region that did not align well with other species. Our *de novo* RNA transcriptome assembly uncovered a full coding transcript for CD47 from devil PBMCs that aligned well with other species analyzed here. Our analysis suggests that the 34 kDa devil CD47 also contains five transmembrane segments and a single Ig-like domain (Figure 8). The four cysteines needed for the disulfide bonds reported in human CD47 are conserved in all species examined here, and overall sequence identity across all species compared here ranges from 54 to 87% (Table 2). Curiously, the ELM tool predicted an ITIM in the devil CD47_LKYHVV_ ECD. All other species except bats and hamsters also contain the putative ITIM domains, but the ELM tool classified the ITIM as an excluded motif due to the putative ITIM being located in ECD of the protein. We also identified a putative TRAF2-binding site in the third devil intracellular domain of CD47_KAVEE_ that was conserved across all species examined here except bats.

### SIRP-α

Devil SIRP-α is a predicted 56 kDa protein. Human SIRP-α binds to CD47 as either a monomer or a dimer (83, 84) and forms three intramolecular disulfide bonds. All six of the cysteines that form intramolecular disulfide bonds in humans are conserved in devils (Figure S1 in Supplementary Material). The percent sequence identity for SIRP-α ranged from 53 to 69%. Analysis of devil SIRP-α revealed two putative ITIM and one ITSM domain. The ITIM_ITYADL_ shares 100% identity with all species except cattle, and the ITIM_LTYADL_ is 100% conserved across all species. The devil ITSM_SQTEYASI_ matches 5/7 amino acids with all other species; to our knowledge no ITSM has previously been reported in SIRP-α. We identified two potential SIRP-β1 orthologs in devils (ENSSHAT00000016950, XM_012553634.1) that contained ITIM motifs (SIRP-β1_VSYNLI_) in the ECD. The putative SIRP-β1 (ENSSHAT00000016950) ITIM aligned with an SIRP-β1_VSYNIT_ sequence in SIRP-α that contains the key tyrosine residue for ITIMs but did not contain the canonical 3′ I/V/L.

### CD200

**Figure 9:**
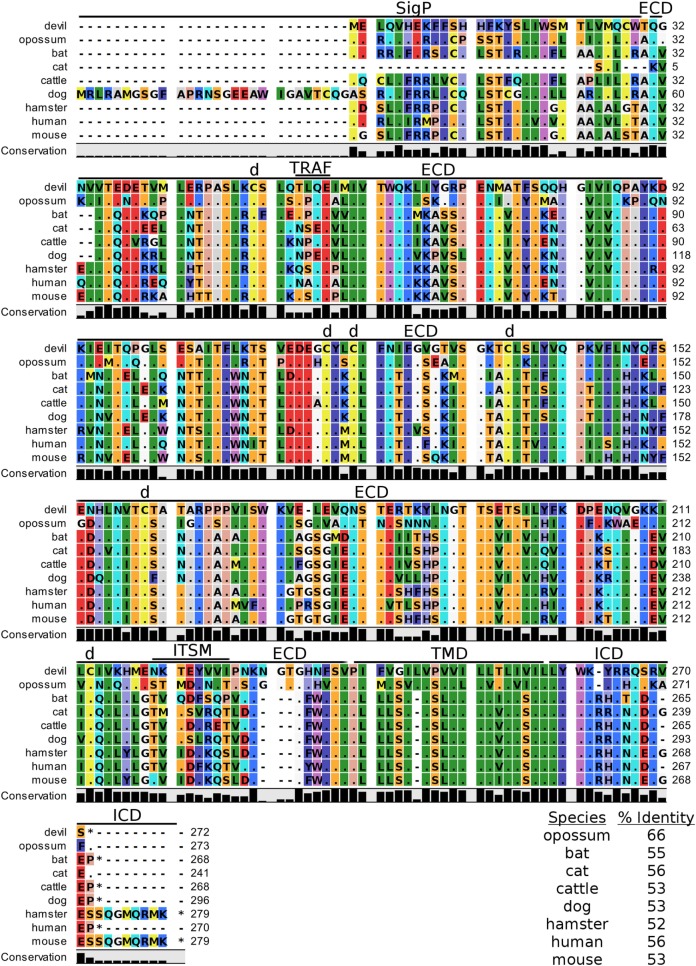
**Alignment of CD200 reference genes for nine species**. The predicted signal peptides (SigP), extracellular domain (ECD), transmembrane domain (TMD), and intracellular domain (ICD) are demarcated with bars. “d” above the alignment represents predicted disulfide bonds. “TRAF” indicates a potential TRAF1/2-binding site and “ITSM” represents a putative immunoreceptor tyrosine-based switch motif (ITSM). The black bar graphs below the alignment represent the conservation of amino acids across all nine species. The percent amino acid sequence identity between devils and other species is shown in the bottom right corner of the alignment.

Devil CD200 is predicted to be a 31 kDa type-I transmembrane protein that shares 52–66% sequence identity across the species compared here (Figure 9; Table 2). The cysteine residues that form intramolecular and intermolecular disulfide bonds necessary for binding as either a monomer or homodimer to CD200 receptor (CD200R) are 100% conserved across all species examined here (Figure 9) (85). Additionally, the Ig-like V-type and Ig-like C2-type domains of human and mouse CD200 were identified by the SMART tool. Many key motifs, including endocytic sorting signals, and TRAF2, SH2, and SH3 binding motifs, are present in all species compared here. Our analysis using ELM predicts a potential ITSM only in devil CD200. However, the devil CD200_TLQE_ TRAF1/2-binding site and CD200_NKTEYVVI_ ITSM are both located in the predicted ECD of devil CD200, so empirical testing is needed to determine if these are functional sites. Although not analyzed in depth here, CD200R and CD200R-like proteins were identified in the nine species compared here. The primary reference sequence for each species contained the key CD200R_NPLY_ motif (86), which has been shown to be a key motif for CD200R inhibitory function (87).

### TIM-3

**Figure 10:**
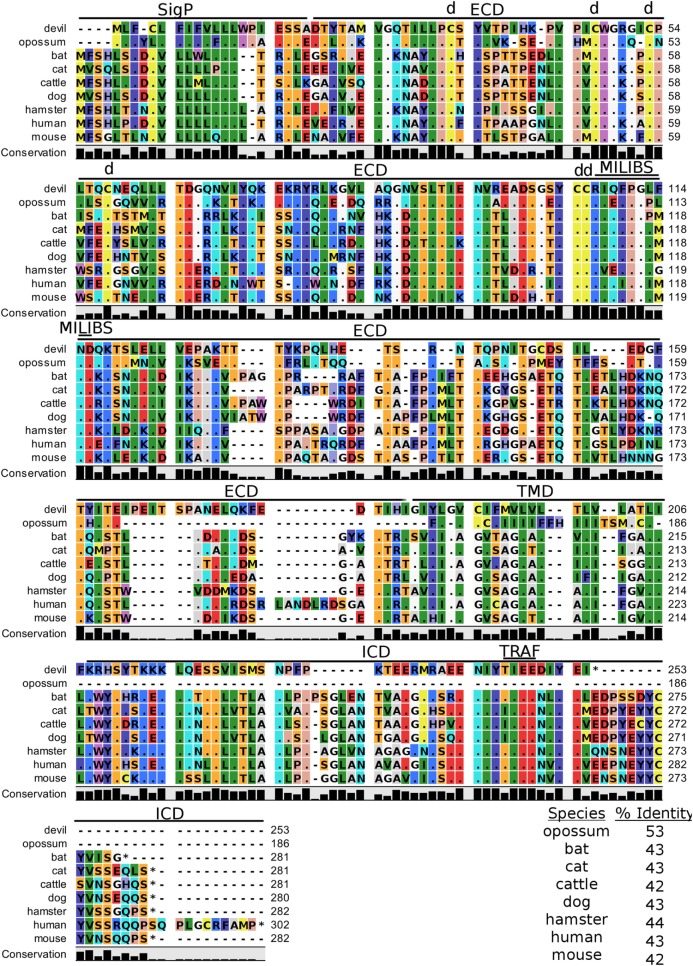
**Alignment of TIM-3 reference genes for nine species**. The predicted signal peptides (SigP), extracellular domain (ECD), transmembrane domain (TMD), and intracellular domain (ICD) are demarcated with bars. “d” above the alignment represents predicted disulfide bonds. “TRAF” indicates a potential TRAF1/2-binding site and “MILIBS” demarcates a conserved metal ion-dependent ligand-binding site (MILIBS) found in phosphatidylserine on the surface of apoptotic cells. The black bar graphs below the alignment represent the conservation of amino acids across all nine species. The percent amino acid sequence identity between devils and other species is shown in the bottom right corner of the alignment.

TIM-3, also known as T-cell immunoglobulin and mucin domain-containing protein 3, hepatitis A virus cellular receptor 2, and CD366, is a surface protein expressed on T cells, Tregs, monocytes, macrophages, and DCs in mice and humans [reviewed in Ref. (88)]. The most up-to-date gene sequences for devil TIM-3 in Ensembl (ENSSHAT00000018107.1) and GenBank (XM_012552117.1) had truncated 3′ region of the coding sequence, and the Ensembl gene had an extended 5′ region that did not align with other species. Our *de novo* RNA transcriptome assembly uncovered a full coding devil TIM-3 transcript from devil PBMCs that aligned well with other species analyzed here. Analysis of the devil TIM-3 predicted sequence suggests that it is a 24 kDa protein with a Ig-like V-type domain and mucin domain in the extracellular region of the protein (Figure 10). Percent amino acid sequence identity between devils and the other eight species analyzed here ranges from 42 to 53%, which is the lowest identity for any molecule analyzed here except for CD80 and CD86 (Table 2). Despite this relatively low conservation of protein sequence, many of the key TIM-3 features identified in mice and humans appear to be present in devil TIM-3.

A unique feature of human TIM-3 is the double cleft which facilitates binding to multiple ligands (89, 90). The six cysteines that form three intramolecular disulfide bonds in the Ig-like region of human TIM-3 (89) are all present in devil TIM-3 (Figure 10), suggesting that devil TIM-3 folding and structure might be similar to human TIM-3. Analysis of human TIM-3 sequences identified five key amino acids for the two ligand-binding clefts; four of the five key amino acids were conserved in devil TIM-3. All species analyzed here also have two critical tyrosine residues in the TIM-3 cytoplasmic domain that align with mouse TIM-3_Y256_ and TIM-3_Y263_ (Figure 10), which are phosphorylated following ligation by GAL-9 and are associated with increased NFAT/AP-1 and NF-κB activity (91). A TIM-3_TIEE_ TRAF1/2-binding site is located between these tyrosine residues in all species except for cats, dogs, and cattle (Figure 10). Also, mouse TIM-1, TIM-3, and TIM-4 all bind to phosphatidylserine on the surface of apoptotic cells *via* a conserved metal ion-dependent ligand-binding site (MILIBS). The mouse TIM-3_RIQFPGLMND_ MILIBS motif is 100% conserved in cats, dogs, and cattle TIM-3, whereas devils have a phenylalanine instead of a methionine in this motif. Whether this TIM-3_M114F_ affects phosphatidylserine binding and clearance of apoptotic cells in devils is unknown.

### Lymphocyte Activation Gene 3

In humans and mice, LAG-3 (CD223) is an inhibitory type-I transmembrane protein that is structurally homologous to CD4 (92). Human and mice LAG-3 each contain one Ig-like V-type and three Ig-like C-type domains, whereas devil LAG-3 is predicted to be a 54 kDa protein with three Ig-like domains. However, a 100 amino acid (LAG3_330–429_) region downstream of the three predicted Ig-like domains could contain a fourth Ig-like domain that was not predicted by the SMART prediction tool (68, 93). Devil LAG-3 percent sequence identity with the other eight species analyzed here ranges from 43 to 73% (Table 2). In humans and mice, the LAG-3_KIEELE_ (Figure S2 in Supplementary Material) motif in the cytoplasmic domain is critical for downstream inhibitory signaling interactions, but this exact sequence is not present in the other species analyzed here. The critical lysine (LAG-3_K468_) in this motif is present in devil (LAG-3_KAEEME_) and all other mammals tested here, with the exception of opossums (LAG-3_IAEQME_).

### B7-H4

**Figure 11:**
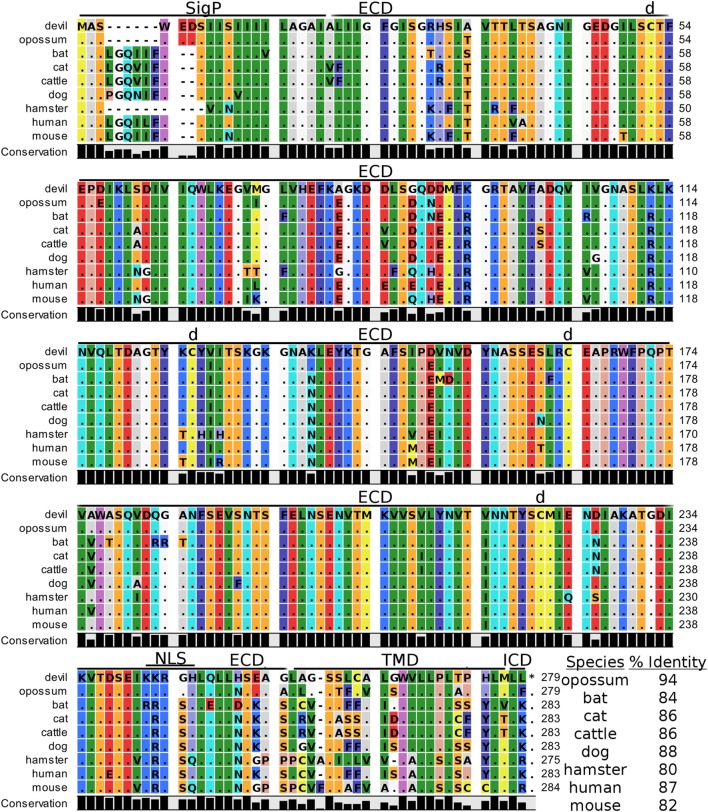
**Alignment of V-set domain-containing T-cell activation inhibitor 1 reference genes for nine species**. The predicted signal peptides (SigP), extracellular domain (ECD), transmembrane domain (TMD), and intracellular domain (ICD) are demarcated with bars. “d” above the alignment represents predicted disulfide bonds. NLS indicates a predicted nuclear localization signal. The black bar graphs below the alignment represent the conservation of amino acids across all nine species. The percent amino acid sequence identity between devils and other species is show in the bottom right corner of the alignment.

Devil V-set domain-containing T-cell activation inhibitor 1, or more commonly referred to as B7 homolog 4 (B7-H4), is a 30 kDa type-I membrane protein that contains two Ig-like V-type domains in the extracellular region (Figure 11). Devil B7-H4 protein shares 87% sequence identity with human B7-H4 and ranges from 80 to 94% for the other seven species analyzed here (Table 2). Based on homology to human and mouse B7-H4 and analysis of devil B7-H4 using cNLS Mapper (score of 3.4) (94, 95), it is likely that devil B7-H4_KKRGH_ contains a bipartite nuclear localization signal (NLS). Interestingly, B7-H4 has only two amino acids in the cytoplasmic domain when expressed as a surface protein.

## Discussion

### Evolutionary Conservation of Key Protein Regions

Despite an estimated 162 million years of evolution since the last common ancestor of placental and non-placental mammals, most of the checkpoint molecules we analyzed have orthologs in Tasmanian devils. Importantly, many of the key signaling motifs and ligand-binding sites are highly conserved in the genes analyzed, particularly cysteine residues for intra- and intermolecular disulfide bonds and transmembrane and intracellular tyrosine residues for receptor tyrosine kinases. The strong conservation of the key protein regions suggests that inhibitory and stimulatory function of these molecules, which is relatively well-documented in humans and mice, is conserved in devils. Importantly, by following established procedures and focusing on the checkpoint molecules that have proven most clinically effective in humans, we can rapidly characterize key checkpoint molecules in devils and incorporate potent immunomodulatory agents into our DFT disease vaccine-development efforts.

### The Web of CD28–CTLA-4 Cosignaling Pathways

Our analysis shows for the first time that the key CTLA-4_MYPPPY_ and CD28_MYPPPY_ are conserved in devils. Given that the MYPPPY motif present in chickens facilitates binding to mammalian CD80 and that a single leucine for methionine substitution in pig CTLA-4_LYPPPY_ is sufficient to disrupt binding of porcine CTLA-4 to human CD80 (35, 72, 96), the evolutionary conservation of CTLA-4_MYPPPY_ and CD28_MYPPPY_ in devils suggests that the competitive binding of CTLA-4 and CD28 for CD80/86 should be present in devils.

In addition to the conserved binding motifs, the key CD28_SDYMNN_ stimulatory domain (97) and CTLA_GVYVKM_ inhibitory domains are also conserved in devils, suggesting that the complementary stimulatory and inhibitory functions of CD28 and CTLA-4 are present. The CTLA-4_GVYVKM_ motif, in association with T-cell receptor-interacting molecule (TRIM), regulates surface expression of CTLA-4 (98). The TRIM-mediated recycling of CTLA-4 from the surface provides the means for cell-extrinsic inhibitory function by trans-endocytosis of CD80/86 (99). By trans-endocytosing, or in other words stripping CD80/86 from the surface of an opposing cell (i.e., APCs) and degrading the CD80/86 molecules in the CTLA-4-expressing cell, the potential of costimulation *via* CD80/86 binding to CD28 is eliminated. Whether trans-endocytosis or trogocytosis (100) occurs in devils is unknown. It could be readily tested by developing CD80 transgenic cell lines that have a fluorescent protein, such as green fluorescent protein (GFP), fused to the 3′ tail of CD80. Flow cytometric or immunofluorescence observations of GFP transferred from the transgenic cell line to the CTLA-4 expressing cells would demonstrate that this inhibitory pathway is conserved in devils.

The complex interactions involving CTLA-4 are further complicated by some anti-CTLA-4 monoclonal antibody effects that are mediated through Fc receptor signaling (11, 101, 102). Tregs expressing high levels of CTLA-4 can be killed *via* antibody-dependent cell-mediated cytotoxicity, thus reducing the inhibition of antitumor immunity. This could be a useful tactic for increasing T cell responses against neoantigens in DFT cells, but little is known about IgG isotypes and Fc receptors in devils (103). To our knowledge only a single IgG subclass has been identified in marsupials (103), and the number and type of Fc receptors are unknown. Investigations into devil isotypes and Fc receptors are needed to aid development of potential monoclonal antibody immunotherapies for devils. Additionally, we identified a potential TRAF1/2-binding site in devil CD80 and a TRAF6-binding site in devil CD86. TRAF6 is associated with NF-κB activation and production of IL-6 and IL-8, whereas TRAF2 is associated with IκB-kinases. The binding site for TRAF2 but not TRAF6 is required CD40-mediated antibody class switching (104–106). Specific targeting of CD80 or CD86 by monoclonal antibodies or soluble ligands could help to determine if additional IgG subclasses exist in devils and to understand the potential role of IL-6 and IL-8 in immune evasion by DFT cells.

In addition to binding to both CTLA-4 and CD28 in humans and mice, CD80 binds to PD-L1 with a greater affinity than to CD28, but less than affinity of CD80 for CTLA and PD-L1 for PD-1 (107, 108). In humans, overexpression of CD80 blocks PD-L1 expression by sequestering PD-L1 in the cytoplasm while simultaneously providing costimulation (109). Additional pleiotropic effects of CD80 include its ability to stimulate NK cells through both CD28-dependent and -independent pathways (110–112). As DFT1 cells are poorly immunogenic and do not express MHC-I, the ability to stimulate NK cells using CD80 could provide immunotherapeutic potential.

Soluble CD80 can also effectively block PD-1:PD-L1 interactions and provide costimulation (113, 114). Interestingly, in addition to the full-length devil CD80, we identified a devil CD80 splice variant that lacks a transmembrane domain (TMD) and likely codes for soluble version of this key checkpoint molecule. Humans are also known to express soluble CD80 and soluble PD-L1, and high plasma levels of PD-L1 are associated with poor prognosis in B cell lymphomas (115–117). The anti-PD-L1 monoclonal antibodies we have previously developed could be used to screen devil plasma for soluble PD-L1 and possibly serve as a prognostic test for DFT disease.

### Devil 4-1BB Function Is Predicted to Resemble Human 4-1BB Function

Despite the potential for enhanced immunotherapies targeting the 4-1BB/L pathway, actual progress has been slow, in part due to differences between mice and humans. 4-1BB costimulation in both mice and humans leads to enhanced T cell-mediated immunity, but the effects are contrasting in human and mouse NK cells. In mice, 4-1BB stimulation leads to enhanced NK cell effector mechanisms, but in humans 4-1BB signaling inhibits NK effector activity and reverse signaling through 4-1BBL on tumor cells can induce IL-10 and TNF-α (77, 118). This is potentially mediated by a putative inhibitory motif 4-1BB_LLYIFK_ in the human cytoplasmic domain that resembles an ITIM domain (76, 77). Interestingly, devils have a similar putative 4-1BB_LPYFLK_ inhibitory domain that includes the key tyrosine residue, but opossum 4-1BB does not contain the putative inhibitory motif (4-1BB_LSFLLQ_). Comparative testing of opossum and devil 4-1BB should be able to determine the inhibitory capacity of these motifs. Devil 4-1BB also contains conserved TRAF1/2-binding sites that in humans are necessary for recruiting TRAF1 and TRAF2 and inducing expression of pro-survival genes, including Bcl-2, Bcl-XL, and Bfl-1 and decreasing expression of Bim, a pro-apoptotic molecule (119–121). Altogether, devil 4-1BB has key features that more closely resemble human 4-1BB, and thus devil 4-1BB might stimulate T cell function but inhibit NK cell function.

As expression of 4-1BB on T cells is largely dependent on activation state, combining agonistic anti-4-1BB immunotherapies with stimulatory cytokines presents an opportunity for promoting immune memory. For instance, CD28- or IL-15-mediated upregulation of 4-1BB combined with agonistic anti-4-1BB monoclonal antibodies or recombinant 4-1BBL could promote antigen-independent survival and expansion of memory CD8 T cells (122, 123); this could be critical for long-term memory against repeated exposure to DFT cells in the wild. Also of particular relevance to the DFT disease is that blockade of 4-1BB:4-1BBL pathways leads to prolonged allograft survival for heart (124), skin (124), eye (125), and intestinal (126) tissues in other species.

### To Eat or Not to Eat: Phagocytic Checkpoints

#### CD47

CD47 functions as an identifier of “self” tissue and is expressed on nearly all human and mouse cells. It has become known as the “don’t eat me” signal due to its ability to inhibit phagocytosis following ligation of SIRP-α (CD172a) on phagocytic cells (i.e., APCs, macrophages) (127). Of particular relevance to transmissible cancers, which are both allografts and cancer, CD47 appears to play a crucial role in transplant tolerance. The potent inhibitory signaling capacity of CD47 was clearly demonstrated in a xenotransplant model in which phagocytosis of pig cells by human macrophages was abrogated by transfecting the pig cells with human CD47 (128, 129). Several other studies have confirmed the importance of the CD47:SIRP-α interaction in graft and xenotransplant tolerance (129–132). Interestingly, the intraspecies CD47:SIRP-α interaction and function seems to be conserved in most species, but interspecies compatibility of CD47:SIRP-α is low (133). This suggests that CD47:SIRP-α binding could be a barrier to interspecies transmission of tumors. The closest extant relatives of devils, the Eastern quoll (*Dasyurus viverrinus*) and spotted-tail quoll (*Dasyurus maculatus*), are also a conservation priority, so understanding the role of CD47 in preventing interspecies transmission could prove useful for conservation of quolls.

DFT1 and DFT2 are not xenotransplants in devils, but the CD47:SIRP-α interaction could be a key mechanism of inhibiting phagocytosis and allo-responses against the DFT cells. Interestingly, in rats and mice, CD47 has been implicated in myelin phagocytosis through modulation of CD47:SIRP-α interactions (134) This is an immune evasion pathway that may have been exploited by DFT1, which originated from a Schwann cell and expresses high levels of myelin (37). Despite the nearly ubiquitous expression of CD47 by all cell types, expression levels are highly variable and can be modulated by inflammation (135, 136). High levels of CD47 found on many types of human cancer, including leukemia (137–139), lymphoma (140), and several types of solid tumor cancers (141, 142) and are often associated with poor prognosis. Importantly, blockade of CD47:SIRP-α interactions stimulated enhanced antitumor responses in several mouse models of human cancer (141, 143, 144), and trials of anti-CD47 antagonistic monoclonal antibodies are underway in humans. The MYC oncogene transcription factor binds to the promotors of both CD47 and PD-L1 and expression of both CD47 and PD-L1 can be downregulated by targeting MYC (145), presenting an opportunity to simultaneously target both PD-L1 and CD47.

A drawback of the anti-CD47 monoclonal antibody treatment is that CD47 blockade can lead to anemia due to rapid depletion of red blood cells. However, this risk could potentially be minimized by injection of anti-CD47 mAbs or SIRP-α fusion proteins directly into accessible DFTs (i.e., tumors in the mouth and face) (146). In addition to facilitating cancer cell immune evasion, CD47 also affects T cell and NK cell homeostasis (147, 148). CD47 is suggested to facilitate longevity by reducing clearance by phagocytes (135). Blockade of CD47 could thus decrease memory cell longevity and increase turnover of T cells and NK cells and could be detrimental to long-term DFT immunity in wild devils that could be re-exposed to DFT.

#### V-Domain Ig Suppressor of T Cell Activation

In contrast to the “don’t eat me” signal from CD47 ligation, VISTA can function as an “eat me” signal *via* intercellular homophilic binding that affects clearance of apoptotic cells and immune surveillance by macrophages and T cells (149, 150). VISTA expression is upregulated in response to genotoxic stress in a p53-dependent manner, and homophilic VISTA interactions both in *cis* and *trans* among tumor cells, APCs, and T cells may facilitate tumor cell escape from immune surveillance (149). No polymorphisms in devil p53 have been reported (151), but given the chromosomal rearrangements and greater than 17,000 somatic mutation reported in DFT1 cells (152, 153), it seems probable that p53 would be at least occasionally activated in DFT cells and could induce upregulation of tolerogenic VISTA signaling.

The tolerogenic role of VISTA in humans and mice and the strong conservation of VISTA across the nine species analyzed here (only B7-H4 and CTLA-4 were more conserved) suggest that VISTA may play an important evolutionary role in maintaining the balance between tolerance and immunity in mammals. Despite high sequence identity, the ELM tool unexpectedly predicted a putative TRAF1/2 (VISTA_SIQE_) binding site in the ECD of devil VISTA. TRAF binding sites are most commonly found in TNF receptors, such as CD27, CD40, OX40, and 4-1BB.

Interestingly, two independent research groups first identified VISTA, but each reported contrasting functional effects of anti-VISTA monoclonal antibodies. One study reported that mouse VISTA inhibited T cell proliferation and cytokine production *in vitro*, that antagonistic anti-VISTA monoclonal antibody treatment exacerbated experimental autoimmune encephalomyelitis, and that VISTA overexpression on tumor cells led to reduced antitumor immunity in mice (154). By contrast, a single dose of agonistic anti-VISTA monoclonal antibody completely abrogated graft-versus-host disease in a partial and complete allogenic hematopoietic cell transplantation in mice with mismatched MHC between donor and host (155). This is of particular relevance to DFT, which in most cases likely has mismatched MHC, so blockade of VISTA could prove useful for breaking tolerance to DFT cells. In addition to the role of VISTA in transplant tolerance, early studies suggest that VISTA could play a more general role in immune escape by tumor cells. Several groups have independently demonstrated that VISTA-deficient mice have enhanced antitumor immunity compared to wild-type mice (156, 157). These effects are independent of PD-1 but are amplified in VISTA/PD-1 double knockout mice (158).

#### TIM-3

Like VISTA, TIM-3 plays a role in phagocytosis of apoptotic cells and T cell immunity [reviewed in Ref. (88)]. Ligation of TIM-3 by phosphatidylserine on apoptotic cells induces an “eat me” signal (159–161). The interaction of TIM-3 with its coreceptors has been associated with diverse functional outcomes, including both inhibitory and stimulatory functions. TIM-3 also binds to secreted and surface-expressed galectin-9 (GAL-9, LGALS9) and negatively regulates Th1 immunity and IFN-γ production (162, 163). As DFT1 cells do not naturally express MHC-I or PD-L1 but upregulate both MHC-I and PD-L1 in response to IFN-γ, the potential role of TIM-3 in IFN-γ production warrants further investigation. TIM-3 and PD-1 are often coexpressed on exhausted CD8+ T cells (164) in mice and exhausted TILs in mice and humans (165–168).

In contrast to the inhibitory function of TIM-3 on adaptive T cells, several types of innate immune cells can be activated by TIM-3 ligation (169). Ligation of TIM-3 on mouse and human DCs promotes secretion of inflammatory cytokines (169). TIM-3 is upregulated by TGF-β on tumor-associated macrophages (TAMs) of the M2 phenotype and ligation of TIM-3 leads to increased NF-κB and IL-6 production and is associated with reduced survival in human hepatocellular carcinoma (170). TIM-3 also binds to the pleiotropic alarmin HMGB1 and suppresses antitumor immunity *via* interfering with the ability of HMGB1 to transport tumor-derived DNA to the endosomes of DCs (171). As large DFTs often become ulcerated and/or necrotic, it will be interesting to see if HMGB1 or other alarmins are expressed.

In regard to receptor structure, TIM-3 is unique due to the formation of both *cis*- and *trans*-heterodimers with carcinoembryonic antigen cell adhesion molecule 1. The *cis* heterodimerization is critical for stable surface expression of TIM-3 and its inhibitory function (172, 173). Together, the conserved disulfide bonds and ligand-binding amino acids, suggest that devil TIM-3 function might be similar to humans and mice. Several of the most widely used cell lines in laboratory research, including Chinese hamster ovary cells, Jurkat RMA-S human lymphoma cells, and 3T3 mouse fibroblast cells, express natural ligands that bind to TIM-3 tetramers (89). This presents the opportunity for the use of cross-reactive reagents for devils and other non-traditional study species, but as a cautionary note, it also has the potential to cause confusing results in attempts to understand checkpoint molecule interactions.

### Paired Inhibitory and Stimulatory Receptors

CD47 binds to several coreceptors, including SIRP-γ, THSB1, and THSB2, which induce either activation or inhibition signals (174, 175). An additional SIRP-family protein, SIRP-β1, does not bind to CD47 and no ligand for SIRP-β1 has been identified to date in humans (176). The SIRP-family proteins are hypothesized to be paired receptors that have evolved to reduce pathogen exploitation of conserved motifs found in inhibitory receptors, essentially a decoy target system that facilitates pathogen control. Pathogens that trigger inhibitory signals *via* conserved motifs in inhibitory receptors would thus also trigger activation signals when exploiting the similar motifs in the activating receptor (177, 178). Analysis of devil SIRP-α revealed two predicted cytoplasmic ITIM and one ITSM inhibitory domains, suggesting the inhibitory capacity of the CD47:SIRP-α interaction is conserved in devils and could be an immunotherapy target. The devil SIRP-α ITSM was not detected in any other species, so empirical testing will be needed to clarify the potential for targeting the CD47:SIRP-α pathway in devils.

Like the SIRP-family proteins, members of the CD200R family are hypothesized to have evolved as paired activating and inhibitory receptors. Specifically, herpesviruses, cytomegaloviruses, pox viruses, and adenoviruses inhibit macrophage activation *via* CD200 mimics that bind to CD200R-family members (179–181). In recent years, CD200 has received greater interest as an inhibitory checkpoint molecule associated with antitumor immunity. CD200 is expressed on a wide range of tumors, including lymphomas (182), myelomas (183), leukemias (184), and gliomas (185), and CD200 expression is correlated with expression of epithelial cancer stem cell markers on prostate, brain, breast, and colon cancers (186, 187).

Expression of CD200 on tumor cells has been associated with impaired NK cell function, increased frequencies of Tregs, suppression of memory T cells, decreased CTL activity, and induction of tolerance to allografts (184, 188, 189). Interestingly, CD200 expression has also been associated with Th2-skewed cytokine profiles and prolonged allograft survival (190–193). The transplantable EMT6 mouse breast cancer line expresses low levels of CD200 *in vitro*, but upregulates CD200 following inoculation into immunocompetent mice (194); this is similar to PD-L1, which is often expressed at low levels *in vitro*, but rapidly upregulated in immune active environments. Given the ongoing transmission of DFT for at least 20 years, immunohistochemical analysis of archived DFT tissue samples should determine expression levels of CD200 in the tumor microenvironment, and understanding this potential inhibitory pathway could help direct vaccine-development efforts.

Like devil PD-1, devil CD200 and SIRP-α are predicted to contain ITSM domains capable of regulating TCR signal transduction. ITSMs have been reported for both PD-1 and SIRP-α, but to our knowledge this is the first report of an ITSM in CD200. Interestingly, the CD200 ITSM was predicted only for devils and not any of the other eight species analyzed here, although it is possible that other isoforms of CD200 exist. There have only been a limited number molecules that have been predicted to contain ITSMs: BTLA; 2B4 (CD244); Siglec7; Siglec9; SLAMF1 (CD150); Ly9 (SLAMF3); CD84 (SLAMF5); SLAMF6 (CD352); and SLAMF7 (195, 196). Paired inhibitory and stimulatory receptors are hypothesized to have evolved in response to exploitation of inhibitory receptors by pathogens. Thus, pathogen motifs that utilize the inhibitory receptor will also trigger the stimulatory signal, so it is interesting that the three of the families that contain ITSMs are also predicted to be paired-receptor families (SIRP, CD200, Siglecs). The original ITSM was identified in CD150 and was named “switch-motif” due to its ability to switch from recruitment of SHP-2 to SHIP in the presence of SH2D1A (17, 197). This flexible switching aspect of the paired receptors could be an additional evolved response to exploitation of inhibitory receptors by common pathogens.

### The Unexplored Function of ECD ITIMs and ITSMs

The short cytoplasmic tail of CD200 and known soluble forms in humans suggests that the primary inhibition through CD200 is mediated *via* ligation of CD200Rs. However, as mentioned above we identified a potential inhibitory ITSM in the extracellular region of CD200_NKTEYVVI_. Interestingly, our analysis predicted putative inhibitory motifs in the extracellular region of several checkpoint molecules: devil CD47_LKYHVV_ ITIM near the border of the first extracellular and TMDs; devil SIRP-β1_VSYNLI_ extracellular ITIM; dog CD80_VKYGDL_ extracellular ITIM; and human CD86_IEYDGI_ extracellular ITIM.

To our knowledge, the function of ITIMs and ITSMs has only been explored in intracellular regions of checkpoint molecules. However, despite most checkpoint molecules being considered transmembrane proteins, the majority of checkpoint molecules covered in this analysis are only surface expressed in particular contexts. For instance, CTLA-4 protein can be detected in the cytoplasm prior to T cell activation, but then is transiently moved to the cell surface following activation. Trans-endocytosis of ITIM-containing CD80 (dog) or CD86 (human) could allow the ECD ITIMs to affect intracellular signaling. Also, CD80, CD86, CD200, CD47, all of which were identified to contain extracellular inhibitory domains, are part of multi-receptor or paired-receptor interactions and the potential role of ECD ITIMs and ITSMs may have been overlooked. This is potentially an understudied aspect of molecular and cellular immunology that warrants further investigation.

### The Potential for LAG-3 As an Adjuvant

Although human LAG-3 has a higher affinity for MHC-II than CD4, the inhibitory effects of LAG-3 in humans and mice are thought to be mediated primarily *via* negative regulation of TCR:CD3 signal transduction rather than competition for MHC-II binding (198, 199). In addition to binding to MHC-II, LAG-3 also binds to C-type lectin domain family 4 member G (CLEC4G, LSECTin) and galectin 3 (Gal-3, LGALS3). In mice, Gal-3 expression can suppress CD8 antitumor responses and expansion of plasmacytoid DCs (200). Tumor cell expression of Gal-3 has also been associated with poor prognosis in non-small-cell lung cancer (201), Hodgkin lymphoma (202), and acute myeloid leukemia (203), but the opposite effect has also been reported for breast (204) and gastric (205) cancer.

Of particular interest for devils is the potential for LAG-3 to facilitate anti-DFT immunity. Immunization of mice with LAG-3+ tumor cells, or irradiated tumor cells along with soluble LAG-3-Ig resulted in tumor regression or reduced tumor growth (206). In addition, soluble LAG-3-Ig binding to MHC-II reliably induced DC maturation, which is indicated by upregulation of MHC-II, costimulatory molecules CD40, CD80, CD83, CD86, and IL-12 and TNF-α cytokines (207, 208). In allogenic bone marrow transplant models, LAG-3 blockade abrogates CD8 T cell tolerance to tissue transplants (209), which suggests that LAG-3 blockade could be a target for inducing CD8 T cell responses to allogenic DFT cells. Additionally, because LAG-3 signaling is bidirectional, DFT expressed Gal-3 could inhibit T and NK cell responses *via* direct Gal-3:LAG-3 interactions. Finally, LAG-3 and PD-1 are coexpressed in exhausted T cells in viral infection and on TILs, and blockade of both LAG-3 and PD-1 has been demonstrated to have synergistic effects in mouse models (33, 210–212). Altogether, there is a strong potential for LAG-3 to stimulate anti-DFT immunity due to the potential for overexpression of LAG-3 on tumor cells and/or administration of soluble LAG-3-Ig to serve as an adjuvant, and to use monoclonal antibodies that block LAG-3 on T cells to reduce T cell inhibition.

### Strong Conservation of B7-H4 Protein across All Species

Despite more than a decade of searching, the identity of the B7-H4 coreceptor remains unknown. Further complicating the functional role of B7-H4 protein is that it is commonly expressed in the cytoplasm and nucleus of cancer cells, and nuclear localization of B7-H4 is associated with cell cycle progression and proliferation of cancer cells (95). B7-H4 is upregulated to the cell surface on T and B cells by several mitogens (213, 214) and on monocytes, TAMs, and DCs in response to IL-6 and IL-10 (215, 216).

Despite the receptor for B7-H4 being unknown, the inhibitory function of B7-H4 in regard to T cell responses and apoptosis has been well-documented (213, 214, 217). Binding of B7-H4-Ig to the putative coreceptor inhibits T cell activation, which can be abrogated by monoclonal blockade of B7-H4 (213, 214). B7-H4 protein has been detected on breast, lung, ovarian, prostate, renal cell, and uterine endometrioid cancer cells and is associated with decreased survival [reviewed in Ref. (20, 218)]. The high sequence identity of B7-H4 between humans and devils (87%) suggests that B7-H4 function might be similar in devils, and thus could be used as a prognostic indicator for DFT in addition to the potential for anti-B7-H4 monoclonal antibody immunotherapy.

### BTLA and TIGIT Inhibitory Genes Not Detected in Devils

The molecules described above demonstrated strong homology to other mammals, but another key inhibitory molecule in humans and mice, BTLA4, has not yet been identified in devils. Multiple BTLAs have been identified in bony fish (*Teleosts*), but have not been identified in amphibians or aves (24). Our search of GenBank and Ensembl and also our *de novo* assembly of a transcriptome from devil PBMCs failed to identify a BTLA gene and full coding sequence for the TIGIT gene in devils. Several TIGIT coreceptors are present in the devil genome and three TIGIT transcript variants reported in the opossum genome. Given that TIGIT expression appears to be tightly regulated in humans and mice (219), we believe it likely that a functional TIGIT gene will eventually be detected in devils through targeted laboratory methods (i.e., RACE PCR).

## Conclusion

In addition to the highly conserved motifs in several molecules, we documented strong conservation of cysteine residues for intra- and intermolecular bonds. These remarkable similarities suggest that immunotherapies that have revolutionized human oncology might be translatable into immunotherapies for the DFT disease. The wealth of data on checkpoint molecule expression and function in humans should facilitate understanding of the key immune evasion pathways employed by the DFTs and allow development of vaccines that target these key pathways. However, given the general paucity of reagents for non-traditional study species, innovative new techniques may be needed to assess the potential roles of the putative signaling motifs, and more generally to assess checkpoint molecule function in species other than humans, mice, and rats.

Investigations into the function of checkpoint molecules in devils, dogs, and hamsters may provide insight into the transmissibility of these tumors and uncover potential immunotherapy targets relevant to both human and veterinary medicine. Additionally, our analysis has spawned new questions about the role of inhibitory ITIM and ITSM motifs and paired receptors that could be of highly relevant generally—not only to human medicine. Further insight into the evolution of the immune system might be gained by studying the role of checkpoint molecules in natural disease systems, such as white-nose syndrome in bats and chytridiomycosis in amphibians. Diversifying immunological studies into non-traditional species may facilitate a broader understanding of key immune defense pathways and elucidate general principles that are unlikely to be found when studying only a small number of model organisms.

## Author Contributions

AF collected, analyzed, and interpreted the data; prepared the figures; and wrote the manuscript. NB performed the *de novo* transcriptome assembly, provided intellectual input, and provided critical feedback on the manuscript. AL and GW provided intellectual input on immunological processes and assisted in the drafting of relevant sections of the manuscript. JH provided intellectual input into the overarching framework of the study, preclinical insight into feasibility of immunotherapeutically targeting immune checkpoint inhibition in species other than humans and mice, and critical detailed review of the manuscript.

## Conflict of Interest Statement

AF and GW received funding from an Entrepreneurs’ Programme Research Connections grant with Nexvet Australia Pty. Ltd. (RC50680). Nexvet is a global clinical-stage biopharmaceutical company focused on transforming the therapeutic market for companion animals by developing and commercializing novel biologic therapies. The other authors declare no conflict of interest.
