# Supplementary material for: Comparative Analysis of Immune Checkpoint Molecules and Their Potential Role in the Transmissible Tasmanian Devil Facial Tumor Disease
Source: Front Immunol. 2017 May 3;8:513. doi: 10.3389/fimmu.2017.00513 (PMC5413580; doi:10.3389/fimmu.2017.00513)
Supplement: Supplementary file 1 [file Data_Sheet_1.PDF]

Supplementary methods

Full Title: Comparative analysis of immune checkpoint molecules and their potential role in the transmissible Tasmanian devil facial tumor disease

Running Title: Comparative analysis of immune checkpoint molecules

Andrew S. Flies[1,2*], Nicholas B. Blackburn[1,3], A. Bruce Lyons[4], John D. Hayball[2,5] Gregory M. Woods[1]

[1]Menzies Institute for Medical Research, University of Tasmania, Hobart, Tasmania, Australia
[2]Department of Pharmacy and Medical Sciences, University of South Australia, Adelaide, South Australia, Australia
[3]South Texas Diabetes and Obesity Institute, School of Medicine, University of Texas Rio Grande Valley, Brownsville, Texas, USA
[4]School of Medicine, University of Tasmania, Hobart, Tasmania, Australia
[5]Robinson Research Institute, Discipline of Obstetrics and Gynaecology, School of Medicine, The University of Adelaide, SA, 5005, Australia

[*] Correspondence:
Andrew Flies
andy.flies@utas.edu.au
+61 0362264614

Supplementary methods

De novo assembly RNA-seq data

Transcriptome assembly from PBMC data was conducted with Trinity v2.2.0 (1,2) The decision to use *de novo* alignment over genome-guided was made on the basis that several of the target genes under investigation are incompletely, or not, annotated in the Tasmanian Devil reference genome release from March 2011 (GenBank Assembly ID: GCA_000189315.1) and an unbiased assessment of all target genes was warranted.

A custom BLAST database (3) was created from the *de novo* transcriptome. BLASTN version 2.4.0+ (4) was used to query coding sequences (CDS) obtained from Ensembl (5), database version 87.7, for the target genes of interest, against the assembled transcriptome. When multiple Tasmanian devil protein coding transcripts were identified from Ensembl for a target gene the CDS for each transcript was queried.

When a matching Ensembl gene CDS was not available for a target gene, the gene was queried in the UCSC Genome Browser (Kent et al. 2002; genome.ucsc.edu [accessed Jan. 25, 2017]) to identify potential transcripts from the 'Ensembl Gene Predictions – 86' track (5). This identified transcripts that were not specifically annotated as the target gene but that did show comparative alignment with the target gene in other species through the 'non-Tasmanian Devil RefSeq gene' Genome Browser track produced by UCSC. This transcript ID was then used to obtain a CDS from Ensembl. When a CDS for the target gene was not identified using either method the CDS for the human version of the target gene, obtained from Ensembl, was used. Supplementary Table X lists the Ensembl transcript IDs for the coding sequences used in this analysis, their source and the resulting number of BLASTN results.

The results of the BLASTN analysis were reviewed to identify a matching transcript from the assembled transcriptome. When multiple transcript matches were identified, the significant alignments were manually assessed against the known CDS for Tasmanian Devil and other species, recognizing that other BLASTN matches may be valid transcripts and include splicing variants or partial transcripts. In this way, we were able to manually search for evidence from the assembled transcriptome of support for the target gene CDS used in our comparative alignment analysis.

RNA-seq data has been uploaded to the National Center for Biotechnology Information Sequence Read Archive with the BioProject number PRJNA381841 and title "Tasmanian Devil (Sarcophilus harrisii) RNA-seq in peripheral blood mononuclear cells."

References

1. Grabherr MG, Haas BJ, Yassour M, Levin JZ, Thompson DA, Amit I, Adiconis X, Fan L, Raychowdhury R, Zeng Q, et al. Full-length transcriptome assembly from RNA-Seq data without a reference genome. *Nat Biotechnol* (2011) **29**:644–52. doi:10.1038/nbt.1883
2. Haas BJ, Papanicolaou A, Yassour M, Grabherr M, Blood PD, Bowden J, Couger MB, Eccles D, Li B, Lieber M, et al. De novo transcript sequence reconstruction from RNA-seq using the Trinity platform for reference generation and analysis. *Nat Protoc* (2013) **8**:1494–1512. doi:10.1038/nprot.2013.084

3. Altschul SF, Gish W, Miller W, Myers EW, Lipman DJ. Basic local alignment search tool. *J Mol Biol* (1990) **215**:403–410. doi:10.1016/S0022-2836(05)80360-2
4. Zhang Z, Schwartz S, Wagner L, Miller W. A Greedy Algorithm for Aligning DNA Sequences. *J Comput Biol* (2000) **7**:203–214. doi:10.1089/10665270050081478
5. Aken BL, Achuthan P, Akanni W, Amode MR, Bernsdorff F, Bhai J, Billis K, Carvalho-Silva D, Cummins C, Clapham P, et al. Ensembl 2017. *Nucleic Acids Res* (2017) **45**:D635–D642. doi:10.1093/nar/gkw1104
6. Kent WJ, Sugnet CW, Furey TS, Roskin KM, Pringle TH, Zahler AM, Haussler D. The human genome browser at UCSC. *Genome Res* (2002) **12**:996–1006. doi:10.1101/gr.229102. Article published online before print in May 2002
7. genome.ucsc.edu. Available at: genome.ucsc.edu

Supplementary Table 1. Reference sequences for the nine species used in comparative sequence alignments of checkpoint molecules

Symbol	HGNC ID	Names	NCBI gene ID	devil	opossum	bat	cat	cattle	dog	hamster	human	mouse
CTLA4	HGNC:2505	CTLA-4, CD152	1493	ENSSHAT00000019213	ENSMODT00000020647.2	XM_006082423.2	NM_001009236.1	NM_174297.1	NM_001003106.1	XM_005070583.1	NM_005214	NM_00984
CD28	HGNC:1653	CD28, T44, Tp44	940	ENSSHAT00000004738.1	XM_007501891.1	XM_014461937.1	NM_001009844.1	NM_181004.1	NM_001003087.2	XM_013113684.1	NM_006139.3	NM_007642.4
CD80	HGNC:1700	B71, B7, BB1	941	ENSSHAT00000008723	XM_007493732.1	XM_014457727.1	AB030651.1	NM_001206439.1	NM_001003147.1	NM_001281419.1	NM_005191.3	NM_009855.2
CD86	HGNC:1705	B72, B70	942	XM_012544547.1	XM_007493776.1	XM_014457759.1	NM_001009229.1	XM_005201386.3	NM_001003146	NM_001310555.1	NM_175862.4	NM_019388.3
C10orf54	HGNC:30085	PD1H, VISTA	64115	***DN110944***	ENSMODT00000000395.3	XM_006090287.2	XM_011287353	NM_001080347.1	ENSCAFT00000022797	XM_013113922.1	NM_022153	NM_001159572.1
TNFRSF9	HGNC:11924	CD137, 41BB, ILA	3604	ENSSHAT00000018829	NM_011612	XM_007493046.2	XM_003989564.3	NM_001035336.2	XM_845243.2	XM_005079387.2	NM_001561.5	XM_006102910.2
CD47	HGNC:1682	CD47, CD47	961	***DN23154***	XM_007493611	XM_014456989	XM_019839653	NM_174708	NM_001080721	XM_013116876	NM_001777.3	XM_006521808
CD200	HGNC:7203	CD200, OX2	4345	XM_012546780.1	XM_007493673.2	XM_014460665.1	ENSFCAT00000007794.3	NM_001034620.2	XM_005639479.2	XM_005074761.2	NM_005944.6	NM_010818.3
HAVCR2	HGNC:18437	Tim3	84868	***DN5208***	XM_007474422.1	XM_006089081.2	XM_003981347.2	NM_001077105.2	NM_001254715.1	XM_005071890.2	NM_032782.4	NM_134250.2
LAG3	HGNC:6476	Lag3	3902	ENSSHAT00000018634	NM_008479.2	ENSMLUT00000013791.2	XM_019835814.1	NM_001245949.1	XM_005637438.1	XM_013110290.1	NM_002286.5	XM_007503669.1
VTCN1	HGNC:28873	VTCN1, B7H4	79679	ENSSHAT00000021759.1	XM_007485264.2	XM_006097486.2	XM_006935023.1	XM_010826862.2	XM_005630662.2	XM_013118073.1	NM_024626	NM_178594.3

Supplementary Table 2. Genes for which the CDS was identified through BLASTN search of the *de novo* transcriptome

Gene	Ensembl transcript ID	BLASTN hits from *de novo* transcriptome query	CDS Source
VISTA	ENSSHAT00000010864.1	10	Ensembl - Tasmanian Devil (via UCSC)
CD47	ENSSHAT00000014546.1	3	Ensembl - Tasmanian Devil
HAVCR2	ENSSHAT00000018107.1	1	Ensembl - Tasmanian Devil (via UCSC)
BTLA	ENST00000334529.9	0	Ensembl - Human
BTLA	ENST00000383680.4	0	Ensembl - Human
TIGIT	ENST00000481065.5	0	Ensembl - Human

Supplementary Table 3. Summary of checkpoint molecule structure. SigP = signal peptide, ECD = extracellular domain, TMD = transmembrane domain, ICD = intracellular domain.

Gene	UniProt families and domains	Mol. weight (Da)	SigP	ECD	TMD	ICD	Ext. coef	Structure	Co-receptors
CTLA-4	Ig-like V-type	25163	1-36	37-161	162-184	184-223	26275	Homodimer, soluble monomer	CD80, CD86
CD28	Ig-like V-type	28264	1-16	17-152	153-175	176-220	55850	homodimer	CD80, CD86, B7-H2 (human)
CD80	Ig-like V-type, Ig-like C2-type	35076	1-24	25-237	238-257	258-311	33390	homodimer, monomer	PD-L1, CTLA4, CD28
CD86	Ig-like V-type, Ig-like C2-type	35145	1-21	22-246	247-269	270-310	38890	homodimer, monomer	CTLA-4, CD28
VISTA	Ig-like V-type	32698	1-30	31-192	193-215	216-308	13450	homodimer (cis and trans)	VISTA (cis and trans), additional unknown ligand
4-1BB	TNFR-Cys repeats (4X)	30572	1-25	26-191	192-216	217-277	29335	homodimer, homotrimer, monomer also binds	4-1BBL
CD47	Ig-like V-type	33819	1-18	19-140, 197-207, 258-267	141-163, 175-196, 207-226, 233-257, 268-287	164-174, 227-232, 288-310	35910	monomer	SIRP-α, SIRP-γ, THBS1, THBS2
SIRP-α	Ig-like V-type, Ig-like C1-type, Ig-like C2-type	55804	1-33	34-367	368-390	391-508	45880	monomer, homodimer	CD47
CD200	Ig-like V-type, Ig-like C2-type	31025	1-30	31-239	240-262	263-271	44265	monomer, homodimer	CD200R1, CD200R2
TIM-3	Ig-like V-type, mucin	23516	1-19	20-184	185-207	208-209	23420	monomer, heterodimer with CEACAM1	CEACAM1, GAL-9, HMGB1, PtdSer
LAG-3	Ig-like V-type, Ig-like C-type (3X)	53885	1-22	22-428	429-450	451-488	89670	homodimer and oligomers	MHC-II, GAL-3, CLEC4G
B7-H4	Ig-like V-type 1, Ig-like V-type 2	29921	1-20	21-256	256-276	277-278	36690	monomer	unknown
PD-1	Ig-like V-type	26924	NA	1-140	141-163	164-243	34170	monomer, homodimer	PD-L1, PD-L2
PD-L1	Ig-like V-type, Ig-like C2-type	30021	1-19	20-236	237-256	257-264	47120	monomer, homodimer	PD-1, CD80
PD-L2	Ig-like V-type, Ig-like C2-type	31217	1-19	20-233	234-256	257-272	34630	monomer, homodimer	PD-1, RGMB

Supplementary Figure 1. Alignment of SIRP-α reference genes for nine species. The predicted signal peptides (SigP), extracellular domains (ECDs), transmembrane domains (TMDs), and intracellular domain (ICDs) are demarcated with bars. ‘d’ above the alignment represents predicted disulfide bonds. “ITIM” and “ITSM” marks putative immunoreceptor tyrosine-based inhibitory motifs and an immunoreceptor tyrosine-based switch motif. The black bar graph below the alignment represent the conservation of amino acids across all nine species. The percent amino acid sequence identity between devils and other species is shown in the bottom right corner of the alignment.

Supplementary Figure 2. Alignment of LAG-3 reference genes for nine species. The predicted signal peptides (SigP), extracellular domains (ECDs), transmembrane domains (TMDs), and intracellular domain (ICDs) are demarcated with bars. ‘d’ above the alignment represents predicted disulfide bonds. “KAEEME” represents a putative inhibitory motif in devil LAG-3. The black bar graph below the alignment represent the conservation of amino acids across all nine species. The percent amino acid sequence identity between devils and other species is shown in the bottom right corner of the alignment. See Table 2 for % sequence identity between devils and other species.
