# Supplementary figures and images for: Comparative Analysis of Immune Checkpoint Molecules and Their Potential Role in the Transmissible Tasmanian Devil Facial Tumor Disease
Source: Front Immunol. 2017 May 3;8:513. doi: 10.3389/fimmu.2017.00513 (PMC5413580; doi:10.3389/fimmu.2017.00513)

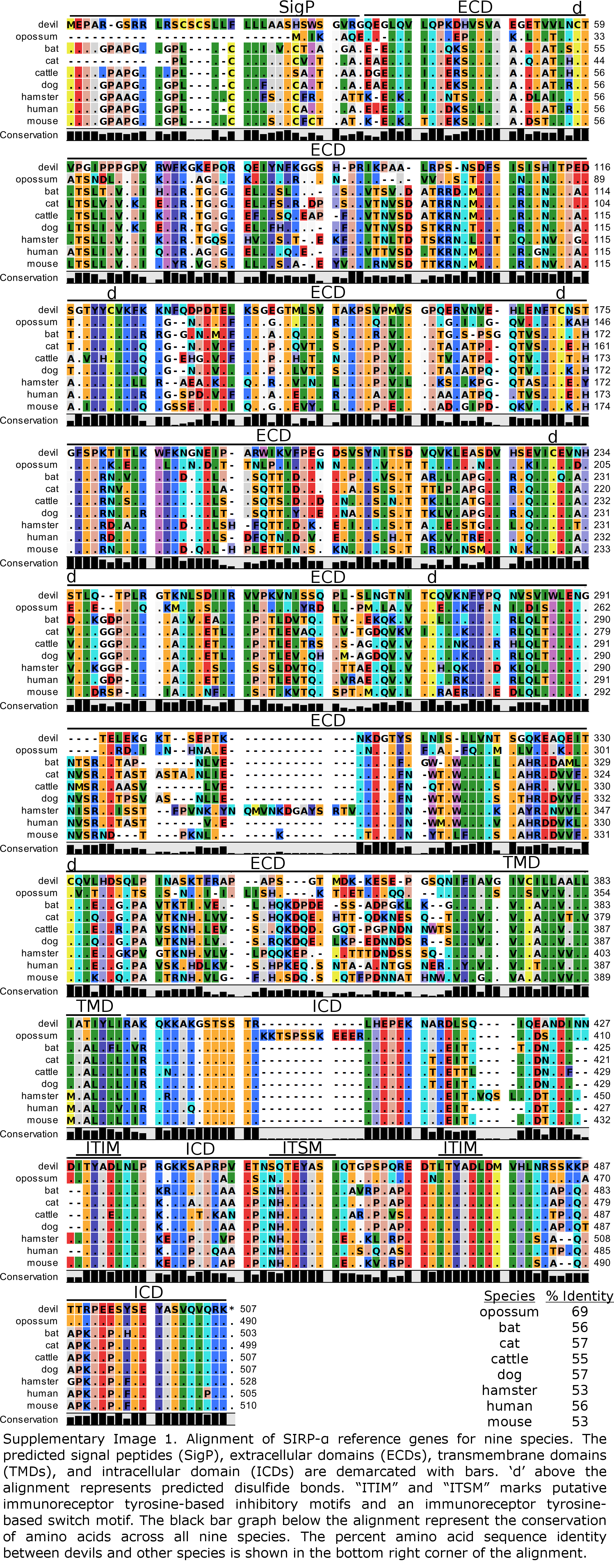

Supplement: Supplementary file 2 [file Image_1.TIF]

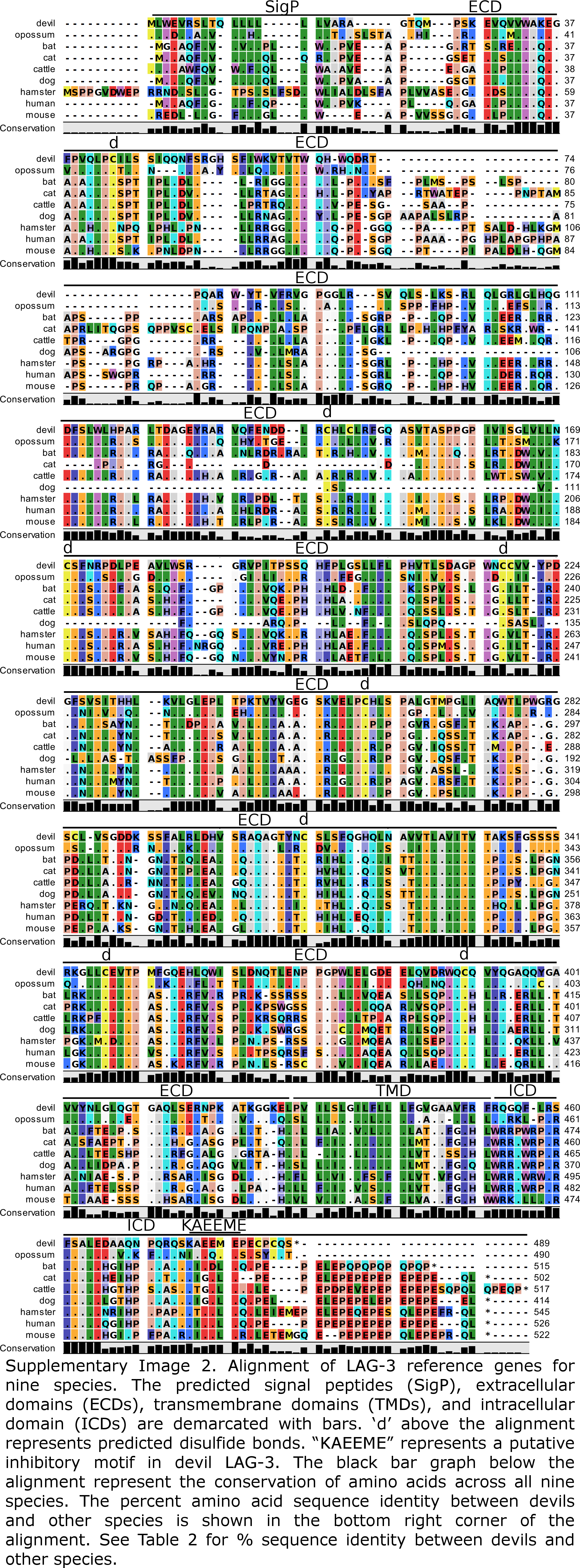

Supplement: Supplementary file 3 [file Image_2.TIF]
